# Network pharmacology, gut microbiota, and metabolomics integration reveal that Shenshuai 'an enema prescription (SSAEP) improves CRF mechanisms by reshaping the gut microbiota and correcting host–microbe cometabolism

**DOI:** 10.1186/s13020-026-01484-z

**Published:** 2026-11-13

**Authors:** Shilin Liu, Andong Li, Ailing Jia, Qiang Chen, Zheng Nan, Zhidong Qiu, Ye Qiu

**Affiliations:** 1 Changchun University of Chinese Medicine, Changchun, 130117 China; 2 Affiliated Hospital to Changchun University of Chinese Medicine, Changchun, 130021 China

**Keywords:** Chronic renal failure, Metabolic profiling, Intestinal microflora, Gut–kidney axis, Shenshuai 'an enema prescription, Network-based pharmacological analysis

## Abstract

**Background:**

Chronic renal failure (CRF) is a global health issue, and the “gut–kidney axis” plays a key role in its pathogenesis. Shenshuai 'an enema prescription (SSAEP) is a Chinese medicine compound based on the TCM theory of “regulation of bowel movement and removal of turbid qi”, which has shown good adjunctive therapeutic effects in delaying the progression of CRF in clinical practice. However, its specific mechanisms remain unclear.

**Purpose:**

Herein, from the perspective of indirect pharmacology, the therapeutic effects of SSAEP on CRF rats were explored. We hypothesize that its mechanism of action may be largely indirect and occurs primarily through the gut–kidney axis. This study aimed to systematically elucidate the potential mechanism through which SSAEP improves CRF through the regulation of gut microecology using multidisciplinary approaches such as network pharmacology, 16S rRNA gene sequencing, and metabonomics.

**Methods:**

A CRF rat model was established by induction with 2.5% adenine. Low and high doses of SSAEP were administered via enema, and antibiotic intervention and fecal microbiota transplantation (FMT) experiments were performed to verify the key mediating role of the gut microbiota. The chemical composition of the SSAEP and the metabolic profiles of the serum and colonic contents of the rats were analyzed using UHPLC‒MS/MS; the composition of the gut microbiota was analyzed through 16S rRNA sequencing; and the core targets and pathways were predicted using network pharmacology and molecular docking. In vitro, human renal tubular epithelial cells (HK-2) were stimulated with TGF-β1 or glycochenodeoxycholic acid (GCDCA) to verify the anti-inflammatory and antifibrotic effects of quercetin (the main active component of SSAEP) and the role of the IL-17 signaling pathway in this process.

**Results:**

SSAEP treatment significantly improved renal function indicators (BUN, SCR, Cys-C, and UA) in CRF rats and alleviated pathological damage, the inflammatory response, and fibrosis in kidney tissue. Antibiotic intervention attenuated the protective effect of SSAEP, while FMT experiments confirmed that the efficacy of SSAEP could be transferred to recipient rats through the transplantation of treated fecal microbiota. SSAEP reshaped the gut microbiota, significantly increasing the abundance of *Akkermansia* while reducing that of *Clostridium*. Metabolomic analysis revealed CRF-related metabolic disturbances and identified 29 key differentially expressed metabolites regulated by SSAEP, among which glycochenodeoxycholic acid (GCDCA) was a key pathogenic metabolite. Network pharmacology predictions combined with in vitro experiments confirmed that the quercetin in SSAEP could effectively antagonize the TGF-β1- and GCDCA-induced epithelial‒mesenchymal transition and inflammatory response in HK-2 cells by inhibiting the IL-17/NF-κB signaling pathway.

**Conclusions:**

This study revealed that SSAEP improves CRF through an indirect pharmacological mechanism dependent on the gut microbiota: SSAEP reshapes the balance of the gut microbiota, thereby correcting host–microbe cometabolic disorders (particularly by reducing the level of the pathogenic metabolite GCDCA) and ultimately alleviating renal inflammation and fibrosis through the inhibition of signaling pathways such as the IL-17/NF-κB pathway. This study provides new experimental evidence for the “gut–kidney axis” theory and lays the foundation for the development of CRF treatment strategies based on the regulation of the gut microbiome.

**Graphical Abstract:**

**Figure:**
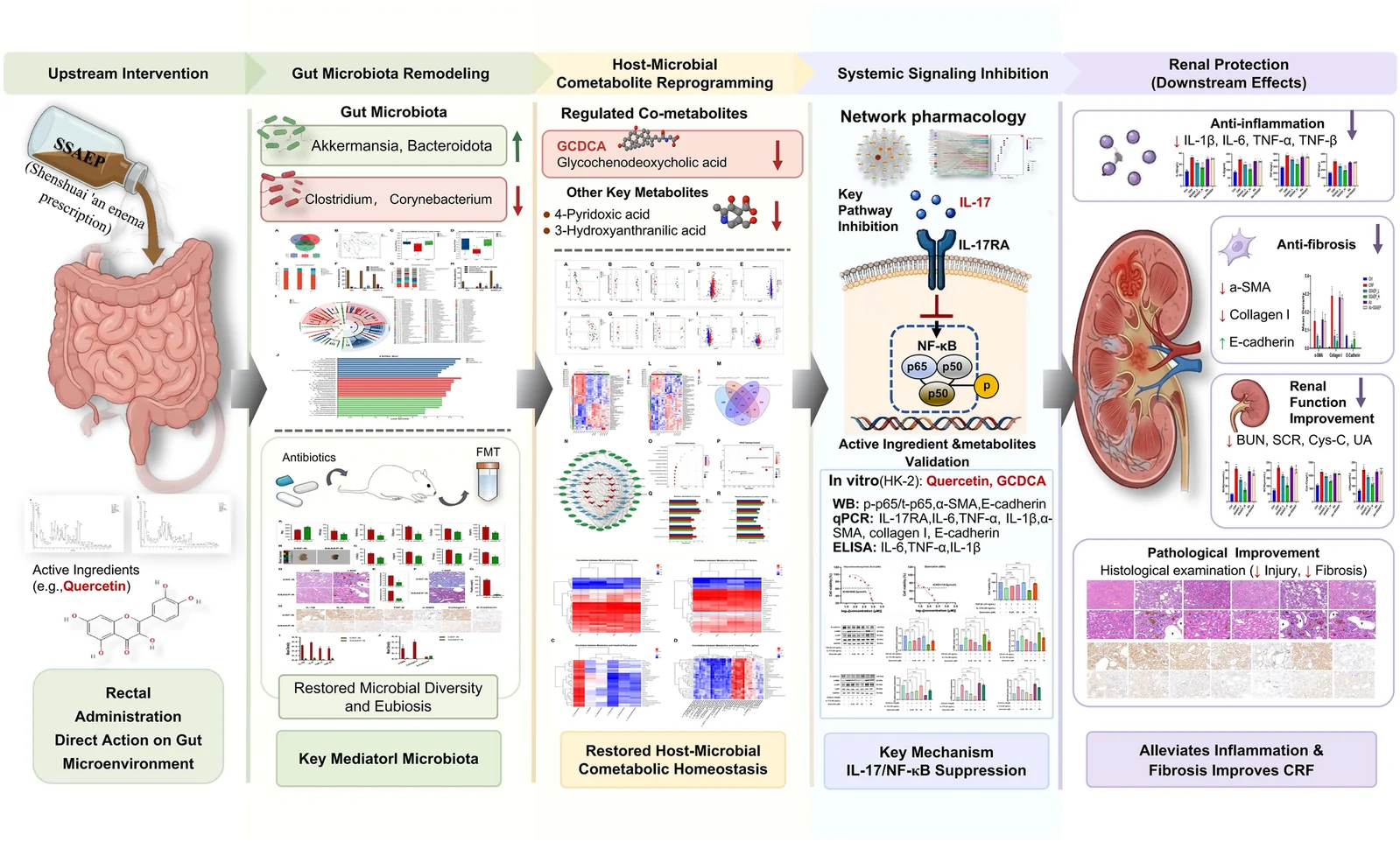

**Supplementary Information:**

The online version contains supplementary material available at https://doi.org/10.1186/s13020-026-01484-z.

## Introduction

Chronic renal failure (CRF) poses a major worldwide health concern [1, 2]. As the latest research shows, CRF progresses slowly and ultimately leads to end-stage renal disease (ESRD) requiring kidney transplant surgery or hemodialysis therapy [3]. Almost 850 million people suffer from kidney diseases worldwide [4], and approximately four million need dialysis to survive [5]. By 2050, chronic kidney disease (CKD) is projected to be the fifth most common cause of early mortality worldwide [5]. In China, there were approximately 132.3 million prevalent cases in 2017 according to a national prevalence survey [6]. Notably, many of these individuals are symptomless until later in the progression of the disease and they often go undiagnosed until it reaches a more severe form (e.g., ESRD), thus missing opportunities for timely treatment of these patients [7]. The treatment and control of fibrosis associated with CRF is difficult, and fibrosis development represents a major challenge for physicians, highlighting a clear need for new strategies that can reduce inflammatory responses and the progression of fibrosis [8–10]. More recent studies have increasingly highlighted the key role played by the intestinal–kidney axis during CRF development [11, 12]. Scientific data have suggested that gut dysbiosis, as well as changes in microbiota-derived metabolites (such as uremic toxins, including indoxyl sulfate and p-cresyl sulfate, and trimethylamine N-oxide (including short-chain fatty acids)), are strongly correlated with systemic inflammation, oxidative stress, and fibrosis of kidney tissues in patients with CRF [13]. Thus, treatments aimed at changing the microbiota composition in the gut and its metabolites may be useful for treating CRF.

One of the advantages of Chinese herbals over synthetic drugs is their ability to treat several targets at one time, which has proven useful in delaying the progression of CRF [14]. Recent research has demonstrated that many TCM prescriptions can improve CRF through the modulation of intestinal microbiota and metabolites in the human body [15, 16]. Shenshuai 'an enema prescription (SSAEP) was formulated by Professor Nan Zheng, a leading authority on TCM for this disease on the basis of his treatment experience according to traditional theories and empirical evidence [17]. This formula has been used with good results in the past half century to treat CRF by applying it as an adjuvant therapy through the rectum, with the proof of concept that it can alleviate disease signs in patients and slow the progression of pathology [18, 19]. It is crucial to clarify that in both our clinical practice and the present study, the SSAEP is exclusively administered as an enema, not as an oral decoction. This route is a deliberate design based on the traditional Chinese medicine theory of “regulation of bowel movement and removal of turbid qi”, which aims to directly target the gut ecosystem and maximize interactions with the intestinal microbiota. The formula mainly consists of ingredients such as the glabrous greenbrier rhizome, milkvetch root, common oyster shell, honeysuckle, wine-treated rhubarb, magnolia officinalis, immature bitter orange, and salted aconite root. Recently, Liu and colleagues reported that the formula may have therapeutic efficacy via anti-inflammatory and antifibrotic effects [20]. However, we do not yet fully understand how it works, including its effects on the gut microbiota and metabolism of CRF patients.

This study systematically explored the impact of SSAEP on CRF and its proposed mechanism of action from the perspective of indirect pharmacology. We hypothesize that SSAEP exerts a renoprotective effect primarily through remodeling the gut microbiota ecosystem. We acknowledge that while this “indirect” hypothesis is strongly supported by FMT and antibiotic intervention experiments, direct pharmacokinetic (PK) evidence is needed to confirm whether the active components reach the kidney at pharmacologically relevant concentrations. This limitation is addressed in the Discussion section.

## Methods and materials

### Reagents

Adenine powder (A800685-100 g, purity ≥ 99.5% as determined by HPLC), vancomycin (V871983-5 g, USP grade, minimum potency of 950 μg/mg), neomycin sulfate (N799581-25 g, 97%), metronidazole (M813526-25 g, 99%) and ampicillin (A830931-25 g, 98%) were purchased from Macklin Company. Histochemical kits were purchased from Wuhan Baiqiandu Biotechnology Co., Ltd., including hematoxylin–eosin stain (B1002), Masson’s trichrome stain (C2110), differentiation reagent (C2105), blueing agent (C2104), EDTA-based antigen retrieval solution (pH 8.0 (C1050) and pH 9.0 (C1051)), citrate buffer antigen retrieval solution (pH 6.0), phosphate-buffered saline (PBS, C0003), hematoxylin (C3876), H_2_O_2_ (C5101), BSA (C5003) and a DAB chromogenic substrate kit (C1001). Common laboratory chemicals such as absolute ethanol (10009218/100092683), xylene (10023418), HCl (10011018) and ammonia, as well as neutral resin 10004160 and neutral resin 10002108, were provided by China National Pharmaceutical Group Chemical Reagent Co., Ltd. The solvents required for liquid chromatography‒mass spectrometry analysis, including chromatographically pure methanol, formic acid, propanol, acetonitrile, and water, were purchased from Fisher Scientific. Shanghai Yuanye Biotechnology Co., Ltd. supplied LC‒MS-grade 2-amino-3-(2-chlorophenyl) propanoic acid as an internal standard/reference material. ELISA kits to detect rat cytokines and biomarkers, including interleukin-6 (IL-6) (MM-0190R2), tumor necrosis factor-β (TNF-β) (MM-0132R2), interleukin-1β (IL-1β) (MM-0047R2), tumor necrosis factor-alpha (TNF-α) (MM-0180R2), and cystatin C (Cys-C) (MM-0438R2), were obtained from Enzyme Immunoassay Biological Co. The reagents used in the 16S rRNA sequencing were as follows: a FastPure Stool DNA Isolation Kit (YH-feces, magnetic bead method, T10-100) for the extraction of genomic DNA from fecal samples was purchased from Shanghai MJYH Corporation; agarose gel (No. 75510019) was purchased from Thermo Fisher Scientific (USA); and fast high-fidelity DNA polymerase (FastPfu^™^) was purchased from Quanshijin Biotech Co. (Beijing). The primary antibodies used in the IHC included tumor necrosis factor-alpha (TNF-α, BA0131), a-SMA (BM0002) (Boster Biological Technology), interleukin-6 (IL-6, GB11117), interleukin-1 beta (IL-1β, GB11113), and collagen type I (GB11022-3), which were purchased from Wuhan Servicebio Technology; epithelial cadherin (E-cadherin, FNab02617) was purchased from Fine Test Biotechnology. The TNF-β (DF6453) reagent was purchased from Affinity Corporation. Secondary antibody conjugates of horseradish peroxidase (HRP) were purchased from the SeraCare Corporation. Rabbit anti-goat IgG-HRP (5220–0362), goat anti-rabbit IgG-HRP (5220–0336), goat anti-mouse IgG-HRP (5220–0341), goat anti-rat IgG-HRP (5220–0364), and the general-purpose secondary antibody HRP-conjugated goat anti-rabbit/mouse IgG (K5007) were obtained from DAKO Corp. The Cell Counting Kit-8 (CCK-8, M96500) required for in vitro experiments was purchased from Invigentech (USA). Human renal tubular epithelial cells (HK-2, JY168) were purchased from Jinyuan Biotechnology (China). Quercetin (B20527) and glycochenodeoxycholic acid (B24724) were sourced from Yuanye Biotechnology (China). Anti-phospho-NF-κB p65 (p-p65, 80379-2-RR), anti-NF-κB p65 (t-p65, 66535-1-Ig), anti-α-SMA (14395-1-AP), anti-E-cadherin (98123-2-RR), prestained protein marker (10–180 kDa, PL00001), and HRP-conjugated goat anti-rabbit IgG (SA00001-2) were purchased from Proteintech (Wuhan, China). The human IL-6 ELISA Kit (MM-0049H2), human TNF-α ELISA Kit (MM-0122H2), and human IL-1β ELISA Kit (MM-0181H2) were sourced from Meimian (China). 2 × Taq PCR Master Mix (RR901A) and the PrimeScript RT Reverse Transcription Kit (RR037A) were purchased from Takara (Japan).

### Herbal extract preparation

The SSAEP consists of 8 kinds of TCMs (Table 1). The raw material was purchased from the Pharmacy Department of the Affiliated Hospital to Changchun University of Traditional Chinese Medicine, and its quality control standard was based on the “Pharmacopoeia of the People’s Republic of China” (Standard No. SYZ-QB-0004–2019). Preparation: The *common oyster shell* and *salted aconite root* were cut into pieces (110 g), which were mixed with 1100 mL of purified water (10:1), gently boiled for thirty minutes, and filtered. An exact amount of filtrate was removed from the graduated beakers, after which it was diluted further with distilled water to make the total volume to 3.9 L. Moreover, the remaining herbs (400 g in total) were soaked for 30 min and added to the decocted water. The mixture was then boiled for 30 min and then filtered with sterile gauze to prepare the liquid; 3000 mL of distilled water was added to the residue, which was subsequently boiled for 30 min, after which it was filtered. All the filtrates were collected together and concentrated by a rotary evaporator, yielding 133 g of concentrate for further processing, after which the dilution factor was calculated (3.83 g of herbs for 1 g of concentrate).

**Table 1:** The composition of the SSAEP includes eight distinct Chinese herbal medicines

Latin name	Chinese name	Herbal name	Medical part	Mass(g)	Batch number
*Rhizoma Smilacis Glabrae*	Tu Fu Ling	*Glabrous greenbrier rhizome*	Dried rhizome	100	20,250,811
*Hedysarum multijugum Maxim*	Huang Qi	*Milkvetch Root*	Dried root	50	20,250,729
*Ostreae Concha*	Mu Li	*Common Oyster Shell*	Conch	50	20,250,702
*Lonicerae japonicae flos*	Jin Yin Hua	*Honeysuckle*	Dried Flower Buds	20	20,250,818
*Radix Rhei et Rhizoma*	Jiu Da Huang	*Wine-treated Rhubarb*	Dry the Roots and Rhizomes	10	20,250,415
*Magnolia officinalis Rehd. et Wils*	Hou Po	*Magnolia officinalis*	Dried Bark	10	20,250,720
*Aurantii fructus immaturus*	Zhi Shi	*immature Bitter orange*	Dried Young Fruits	10	20,250,721
*Radix Aconiti Lateralis Preparata*	Hei Shunpian	*Salted aconite root*	Dried root	5	20,250,816

### Chemical composition analysis of the SSAEP using UHPLC‒MS/MS technology

An appropriate volume of the SSAEP sample was transferred into a 2-mL centrifuge tube, and an appropriate amount of analytical-grade methanol was added. Both solutions were mixed thoroughly using a vortex mixer for 10 min before they were centrifuged at 12,000 rpm for 10 min at 4 °C in a thermostatically controlled centrifuge. After centrifugation, the supernatant was filtered through a 0.22-µm filter. The filtrate was supplemented with an internal standard (2-chlorophenylalanine), which was added at a concentration of 100 µg/mL, resulting in a final concentration of 2 mg/L. Liquid chromatography‒mass spectrometry (LC‒MS) was performed using a Thermo Vanquish high-performance liquid chromatography (HPLC)/Q Exactive HF Orbitrap mass spectrometer. A sample volume of 2 µL was introduced using an automated injector kept at 4 °C, and separation was carried out using a reversed-phase C18 (Zorbax Eclipse C18, dimensions (1.8 µm × 2.1 mm × 100 mm)) with a column temperature of 30 °C, solvent flow rate of 0.3 mL/min, mobile phase A consisting of 0.1% v/v formic acid in water and mobile phase B consisting of 0.1% v/v formic acid in acetonitrile. The flow was delivered in a gradient program as follows: 5% B from 0 to 0.5 min; the proportion of the B-phase was increased gradually to 30% from 0.5 to 3 min, kept stable between 3 and 3.5 min, then increased linearly from 3.5 to 6 min; the amount of B-phase was increased linearly from 5 min (56%) to 7 min (78%) and then kept constant until 8.5 min; the B-phase fraction was then further increased to 95%, and the peak value of 95% was maintained at 8.5–10 min. In the next 0.1 min (between 10 and 10.1 min), the B-phase was quickly decreased back to the initial 5% and maintained from 10.1 to 11 min. Detection by mass spectrometry was performed in both polarities, positive and negative, using an electrospray ionization (ESI) interface throughout the analysis.

### Development and manipulation of the CRF animal model

Specific pathogen-free (SPF) grade male Sprague–Dawley rats (SD) at the postnatal age of 35 d weighing 130 ~ 170 g were used as models and were purchased from Liaoning Changsheng Biotechnology Company (Laboratory Animal Use License No. SCXK (liao) 2020–0001). All animal experiments were conducted in strict accordance with the Guide for Methodology of Pharmacology Research in Traditional Chinese Medicine [21–23], and were conducted in accordance with the appropriate regulations for laboratory animal care. This study was approved by the Animal Ethics Committee, Changchun University of TCM (Ethical No. 2025577). During the entire study period, all the rats were kept at Changchun University of TCM Animal Center (Laboratory Animal Facility License No. SYXK (Ji) 2023–0015), where the conditions were controlled to some degree with respect to light (12-h light/dark cycle), temperature (20–24 degrees Celsius) and humidity (40–70%). Following an acclimation period of one week, the animals were randomly assigned to one of two groups, namely, a control group (Ctrl group, baseline, n = 10) or a model group (n = 50). The control group was given 2 ml of pure water by enema each day. The model group rats were orally administered 2.5% adenine water at a dosage of 200 mg/kg/d until 3 weeks later as a method of inducing CRF [21–23], as continuous administration of adenine can induce CRF in animals.

After the animal model was established, all the animals were divided randomly and evenly into five treatment groups (n = 10) with the aid of a random number table. The CRF group included rats treated with 2 ml of distilled water per day via the rectum; the SSAEP_L group included rats treated with 2 ml of herb extract through the rectum every day at a dosage of 1.5 g/kg/day; the SSAEP_H: group rats were given 2 ml of herb extract by rectum every day at a dosage of 4.5 g/kg/day; the Ab group of animals received broad-spectrum antibiotics in the drinking water twice a week as well as a 2-ml distilled water enema per day; the Ab + SSAEP group was given the same drug treatment plus the administration of a 2-ml herbal extract enema containing 4.5 g/kg/day for 28 days. All the animals underwent an overnight fast at the end of treatment, and their final body weight was measured prior to euthanasia by cervical dislocation and collection of blood, kidney and gut contents for further processing. All the samples were stored immediately after collection at −80 °C for subsequent analyses. All the animals were disposed of by incineration according to our institutional guidelines under proper environmental conditions and standards in an appropriate animal facility.

Enemas were administered via a nonpuncturing syringe connected to an 8-cm-long, 8-gauge silicone catheter (the enema tube was inserted to a depth of 8 cm; after completion of slow injection, the rat was placed in a head-down position, and gentle pressure was applied to the anus for 2 min). Aqueous solutions of antibiotics were prepared by dissolving 0.5 g of vancomycin, 1 g of neomycin sulfate, 1 g of metronidazole, and 1 g of ampicillin in 1 L of sterile water, followed by ultrasonication to ensure homogeneity.

### Implementation of fecal microbial transplantation (FMT)

In this paper, the fecal samples were collected from the CRF and SSAEP_H groups. All the fecal samples were thawed in a water bath at 37 °C. A diluted liquid was made up of 1 g of feces and 10 ml of sterile saltwater and vigorously vortexed for 5 × 3 min (with a 15-s pause in between). The collected feces were filtered through a 200-μm sieve 3 times to remove fecal debris and prepared into a uniform fecal slurry (100 mg/ml) [24].

For fecal transplantation, fecal recipient adult male Sprague‒Dawley rats were matched for age and body weight to the donor animal and acclimatized for seven days. When the experiment started, the animals were orally administered 2.5% adenine solution for 21 consecutive days (200 mg/kg) until the target disease was established. After successful modeling, an FMT experiment was conducted. To prepare the intestinal environment, the animals were administered a combination of the broad-spectrum antimicrobials neomycin sulfate (1 g/L), vancomycin (0.5 g/L), metronidazole (1 g/L), and ampicillin (1 g/L) in their drinking water for seven consecutive days prior to transplantation [24].

The recipients were then randomly divided into two experimental groups (n = 10), namely, CRF-R (fecal transfer group from CRF donors) and SSAEP-R (fecal transfer group from SSAEP_H donors), receiving one dose per day via rectal instillation of feces collected from their respective donor animals (10 ml/kg) for a total of 14 days. At the end of each experimental procedure, all the rats were fasted overnight, weighed, and killed by cervical dislocation; blood and kidney tissue were taken from the animals. The harvested tissue samples or colon pieces were stored at −80 °C until further analysis. The bodies of dead animals were then sent to a landfill site for burial purposes.

### Evaluation of renal function markers and inflammatory mediators in rat serum

The blood samples collected from the various groups were incubated at room temperature for 2 h, cooled at 2 ~ 8 °C, and then centrifuged at 3000 r/min for 0.5 h to obtain the supernatant. Serum levels of urea nitrogen (BUN), creatinine (SCR), and uric acid (UA) were subsequently measured using automatic biochemistry analyzers. Moreover, tissue and serum levels of Cys-C were detected via ELISA. Similarly, serum levels of proinflammatory cytokines, including IL-1β, IL-6, TNF-α, and TNF-β, were quantified using the same ELISA protocol. The levels of IL-6, TNF-α, and IL-1β in the cell culture supernatants were measured using commercial ELISA kits (Meimian Biotechnology, China) following the manufacturer's instructions.

### Western blotting (WB) and quantitative real-time PCR (qPCR)

Cells were harvested and lysed using RIPA buffer containing protease and phosphatase inhibitors. Total protein was separated by 10% SDS‒PAGE and transferred onto polyvinylidene fluoride (PVDF) membranes. After blocking with 5% nonfat milk, the membranes were incubated overnight at 4 °C with primary antibodies against phospho‑p65 (p‑p65), total p65 (t‑p65), α‑smooth muscle actin (α‑SMA), E‑cadherin, and GAPDH (loading control). The membranes were subsequently probed with horseradish peroxidase (HRP)-conjugated secondary antibodies, and immunoreactive bands were visualized using enhanced chemiluminescence (ECL) detection. Band intensities were quantified using ImageJ software.

Total RNA was extracted from the treated cells using TRIzol reagent and reverse‑transcribed into complementary DNA (cDNA) according to the manufacturer’s instructions. Quantitative real‑time PCR was performed using SYBR Green Master Mix on a LightCycler 480 system with gene‑specific primers for IL‑17 receptor A (IL‑17RA), IL‑6, TNF‑α, IL‑1β, α‑SMA, Collagen I, and E‑cadherin (the primer sequences are listed in Table 2). GAPDH served as the internal control. Relative mRNA expression levels were calculated using the 2⁻ΔΔCt method.

**Table 2:** Sequences of primers used for quantitative real-time PCR (qPCR)

Primer name	Primer sequence
IL-17RA-F	ACCACAGTTCCCAAGCCAGTT
IL-17RA-R	ACAGATCCCACCAGCAGAATG
IL-6-F	CAACGATGATGCACTTGCAGA
IL-6-R	GTGACTCCAGCTTATCTCTTGGT
TNF-α-F5	CCACCACGCTCTTCTGTCTA
TNF-α-R5	CCACTTGGTGGTTTGTGAGTG
IL-1β-F	TGCCACCTTTTGACAGTGATG
IL-1β-R	AAGGTCCACGGGAAAGACAC
α-SMA-F1	AGCCATCTTTCATTGGGATGG
α-SMA-R1	CCCCTGACAGGACGTTGTTA
Col1a1-F6	CTGGCGGTTCAGGTCCAAT
Col1a1-R6	TCCAAACCACTGAAGCCTCG
E-cadherin-F	AACCCAAGCACGTATCAGGG
E-cadherin-R	GAGTGTTGGGGGCATCATCA

### Histological examination

HE staining, Masson’s trichrome staining, and IHC were performed as described below.

Kidney tissues were fixed with 4% formaldehyde solution at least overnight and dehydrated through an increasing series of ethanol concentrations, after which the tissues were fixed in paraffin, sectioned at a thickness of 4 microns, deparaffinized, stained with HE, and then observed under a microscope to obtain images for assessment. The nuclei were stained blue to dark blue under a microscope, the cytoplasmic staining was pink, and erythrocytes were bright pink, with weak red to pinkish colored collagen tissue in the HE-stained sections. For Masson’s trichrome staining, the paraffin-embedded sections were deparaffinized, followed by successive treatment with potassium dichromate, hematoxylin, acid fuchsin, phosphomolybdic acid and aniline blue solution. The sections were stained, differentiated in 1% acetic acid, and mounted in neutral balsam for examination under a microscope. In terms of the images collected by microscopy examination, the contrast of the color of the samples was clear after Masson staining, with the collagen structures appearing blue, whereas the muscle cells, fibrins and red blood cells were red; thus, different parts of the sample were colored to make it easier to see its structure.

The tissue sections were deparaffinized and rehydrated through xylene and graded alcohols prior to IHC staining. The tissues were microwaved for epitope unmasking using a citrate solution (pH = 6.0) contained in a retrieval chamber. To block endogenous peroxidases, 3% H_2_O_2_ was added prior to histochemical staining of regions of interest; 3% BSA was used to block nonspecific antigenic sites, after which the primary antibody was applied, and the secondary antibody was added in succession. Colorimetric detection of the antigen‒antibody complex was performed via DAB reagent, after which the nuclei were counterstained with hematoxylin. The prepared regions were then washed with successive concentrations of ethyl alcohol, transferred into xylene for clearing, and finally immersed in neutral balsam to harden them. When observed under the microscope, the nuclei stained by hematoxylin appeared as blue bodies in reflected light. Under microscopic examination, positive immunoreactivity manifested as distinct brown or dark brown staining patterns.

All histological assessments, including HE staining, Masson's trichrome staining, and IHC, were performed by two independent pathologists who were blinded to the group allocation. All pathological scoring was conducted under the same blinded conditions. For the quantitative analysis of HE staining, a semiquantitative scoring system was employed. The glomerular injury score was evaluated by examining at least 20 randomly selected glomeruli per section using a 0–4 scale (0 = normal, 1 =  < 25% injury, 2 = 25–50% injury, 3 = 50–75% injury, 4 =  > 75% injury). The tubulointerstitial injury score was evaluated on the basis of the percentage of cortical area affected by tubular dilation, atrophy, cast formation, and interstitial fibrosis/inflammation on a comparable 0–4 scale. The percentage of Masson's trichrome-stained collagen fiber area and the mean optical density values for immunohistochemistry were quantified using Image-Pro Plus 6.0 software.

### 16S rRNA gene sequencing analysis of the fecal samples

The total microbial genomic DNA of fecal samples from rats in the Ctrl group (n = 6), CRF group (n = 6) and SSAEP_H group (n = 6) was extracted via a FastPure Stool DNA Isolation Kit (MJYH, Shanghai, China) according to the manufacturer’s instructions. The quality of the extracted genomic DNA was evaluated by determining the integrity of the samples via electrophoresis with 1.0% agarose gels, while the concentrations were determined using a NanoDrop2000 spectrophotometer (Thermo Scientific, USA). The obtained DNA was stored at −80 °C until further processing. The V3-V4 region of 16S rRNA genes was amplified to profile the bacterial communities using the following primers: forward primer 338 F (5'-ACTCCTACGGGAGGCAGCAG-3') and reverse primer 806R (5'-GGACTACHVGGGTWTCTAAT-3') and a thermal cycler PCR machine (T100, Bio-Rad, USA). The PCR mixture (20 μl final volume) contained 10 μl of 2 × Pro Taq master mix, forward and reverse primers (8 µl; 5 µM), template DNA (8 µl; 10 ng/µl) and distilled water to make up the final volume. The amplification program was as follows: preheating at 95 °C for 3 min, followed by repeated steps of 95 °C (denaturation) for 30 s, annealing for 30 s, and extension at 72 °C for 45 s; the final extension step was performed at 72 °C for 10 min, followed by holding at 10 °C. PCR products obtained from the same sample were combined and separated by 2% agarose gel electrophoresis. The size of the amplified fragments was verified by electrophoresis on 2% agarose gels, and the amount of the purified product was measured with a Synergy HTX microplate reader (Biotek, USA). The amplicons were subsequently used to prepare next-generation sequencing libraries with the NEXTFLEX Rapid DNA-Seq Kit (Bioo Scientific, USA). The library prep consisted of the following steps: (1) adapter ligation; (2) removal of adapter dimers by magnetic bead-based clean-up; and (3) amplification of the library templates by PCR to increase the amount of template. Finally, the PCR products were purified with magnetic beads and then prepared as the sequencing library. The sequencing was performed by using paired-end reads (300 bp) on an Illumina NextSeq 2000 instrument, with technical assistance from Shanghai Meiji Biomedical Technology Co., Ltd. All the raw sequence data were submitted to the NCBI Sequence Read Archive (SRA), which is freely accessible at PRJNA1413892.

### Metabolomic analysis

Serum samples and colonic content samples were collected from the following groups of mice: Ctrl (n = 6), CRF (n = 6), and SSAEP_H (n = 6). The above biological materials were subjected to metabolomics analysis by LC‒MS/MS. Sample analysis: Sample analysis was performed on a Thermo Fisher UHPLC-Q Exactive HF-X ultrahigh-performance liquid chromatography (UHPLC) mass spectrometer in the laboratory of Shanghai Meiji Biomedical Technology Co., Ltd., Ltd. The separation analysis was performed using a 100 mm × 2.1 mm I.D. ACQUITY UPLC HSS T3 C18 analytical column containing 1.8-µm stationary phase particles. The chromatographic system (Waters, Milford, USA) used mobile phases consisting of two solvents: A-95% aqueous solution + 5% acetonitrile; eluent A consisted of 47.5% acetonitrile:isopropanol (v/v) + 5% water acidified with 0.1% formic acid and eluent B consisted of 47.5% isopropanol + 47.5% acetonitrile + 5% water acidified with 0.1% formic acid; a fixed injection volume of 3 µL was used during the analysis, with a chromatography column temperature of 40 °C. Electrospray ionization (ESI) MS measurements were carried out over the range from m/z = 70 to m/z = 1050 with both polarities on a sheath gas flow of 50 arb and auxiliary gas flow of 13 arb, with an auxiliary gas heater temperature of 425 °C and the ionization source set to + 3500 V for positive mode and −3500 V for negative mode analysis. The ion transfer tube temperature was set at 325 °C, with a stepwise increase in the collision energy range from 20–40 to 60 V applied for the analysis. The mass spectrometer was operated in full-scan MS mode with a resolution of 60,000 and in ion mode with a resolution of 7500 in data-dependent acquisition (DDA) mode. The resulting raw files were analyzed using Progenesis QI version 3.0 (Waters Corporation, Milford, USA) to perform the following analyses: baseline correction, peak detection and integration, retention time alignment between runs, and alignment of the respective maximums. The resulting data contained information on the retention times, m/z, and relative ion intensities. To identify compounds, the acquired MS and MS/MS spectra were compared with several reference libraries: the Human Metabolome Database (HMDB), the Metlin database, and the internal spectral library developed by Meiji. The criteria for identification required a precursor ion accuracy of less than 10 ppm, whereas the secondary fragmentation pattern had to be confirmed via a spectral match algorithm for the metabolites to have a high level of annotation confidence.

### Network pharmacology analysis

The chemical composition of the SSAEP was characterized by UHPLC‒MS/MS. After that, the potential targets of the screened components were predicted by and obtained from the TCM Systems Pharmacology Database (TCMSP). We set the condition such that the predicted values of intestinal epithelial permeability (Caco-2) were ≥ −0.4 nm/s and the drug likeness was (DL) ≥ 0.18 to. For those chemicals not uploaded into the TCMSP database, their molecular structure was obtained from the PubChem compound database and then further analyzed using the online software SwissADME. We selected the following standards to screen out active ingredients: (1) a high intestinal absorption rate; for this screening, we considered only those molecules that passed a minimum of 2 out of all 5 drug likeness filters (Ghose’s rules, Veber’s rules, Egan’s rules and Muegge’s rules). (2) Possible target proteins of the filtered molecules were predicted using the Swiss Target prediction web server (http://www.swisstargetprediction.ch/), where target proteins with a prediction score greater than 0.1 were regarded as potential targets for the active ingredients, and all acquired targets were normalized using the UniProt database (https://sparql.uniprot.org/) to obtain a standard gene name.

To identify CRF-related genes, we conducted a series of extensive literature searches on “chronic renal failure” in GeneCards (https://www.genecards.org/, OMIM (https://www.omim.org/), and DrugBank (https://go.drugbank.com/). Afterward, we cross-checked the obtained CRF-related genes with the predicted targets of SSAEP as potential therapeutic targets. A Venn diagram of the common target genes of SSAEP and genes related to CRF was constructed to visualize the overlap. The relationships between the bioactive compounds and targets of SSAEP were displayed using Cytoscape (v3.10.2). The importance of each node within the network was assessed by topology analysis, where the symbols represent centrality, connection strength, etc. A protein interaction network (PPI) was constructed using the String database (https://stringdb.org/). Functional annotation and pathway analysis were performed via the Metascape website (https://metascape.org/), including GO enrichment and KEGG pathway enrichment analyses, where the significance level was set to *P* < 0.05.

### Molecular docking

Through the PPI network analysis, several candidate target molecules were screened and given higher priorities, which we further investigated using molecular docking experiments. We downloaded 3D protein structures for relevant target gene proteins from the PDB database. Moreover, the 3D structures of major active ingredients were obtained from PubChem, and the file format was standardized by converting the molecular structure files into a consistent format through the Open Babel program., AutoDockTools software was subsequently used for docking simulation calculations of the receptors and ligands. The interaction strength of the molecules was judged on the basis of their binding free energy, where higher absolute values represent more stable molecular complexes. Finally, we visualized the docking output using 3D and 2D representations and analyzed its conformation with PyMOL and Discovery Studio 2025 software.

### In vitro cell culture and functional validation

Human renal proximal tubular epithelial cells (HK-2) were obtained from Jinyuan Biotechnology (Nanjing, China). Cells were cultured in Dulbecco's modified Eagle medium (DMEM; Gibco, USA) supplemented with 10% fetal bovine serum (FBS) and 1% penicillin/streptomycin at 37 °C in a humidified atmosphere containing 5% CO₂. Cell viability was assessed using a Cell Counting Kit-8 (CCK-8; Invigentech, USA). HK-2 cells were seeded in 96‑well plates at a density of 5 × 10^3^ cells per well. Following the indicated treatments, 10 μL of CCK-8 solution was added to each well, and the plates were incubated for 1.5 h. Absorbance was measured at 450 nm using a microplate reader.

In the quercetin validation experiment (n = 3), the cells were allocated to seven experimental groups: Group 1: Control (PBS); Group 2: TGF‑β1 (10 ng/mL) (Model); Groups 3–5: TGF‑β1 + Quercetin (6. 25, 25, and 50 μM); Group 6: TGF‑β1 + IL‑17A (50 ng/mL); Group 7: TGF‑β1 + IL‑17A + Quercetin (50 μM). All treatment regimens were maintained for 48 h.

For the GCDCA validation experiment (n = 3 per group), the cells were divided into seven groups: Group 1: Control (PBS); Group 2: GCDCA (150 μM) (Model); Groups 3–5: GCDCA + Quercetin (6. 25, 25, and 50 μM); Group 6: GCDCA + IL‑17A (50 ng/mL); Group 7: GCDCA + IL‑17A + Quercetin (50 μM).

Cells were pretreated with quercetin for 1 h before the addition of GCDCA alone or in combination with IL‑17A. The total treatment duration was 24 h.

### Statistical analysis

The determination of the microbial profile based on 16S rRNA high-throughput sequencing, as well as the detection of metabolites by liquid chromatography‒mass spectrometry (LC‒MS) nontargeted metabolomics, was carried out using the Majorbio Cloud Platform (https://cloud.majorbio.com), while the levels of related inflammatory factors and collagen fibers in kidney tissues were detected by hematoxylin‒eosin (HE) staining and Masson’s trichrome staining, respectively. We performed our statistical analyses with the software package IBM SPSS Statistics v.27, and for plotting our figures, we employed GraphPad Prism 10.1.1. We report the results as the arithmetic *means* ± *standard deviations* ($\overline{x} \pm s$) while comparing multiple experimental conditions by either *one-way analysis of variance* or the *Kruskal‒Wallis test* according to whether the data were normally distributed or not. In the FMT trial, we compared groups with *Student’s t test* (for normally distributed data) or the *Wilcoxon rank-sum test* depending upon whether the assumption of normality of the distribution of the data with homogeneous variance held true or otherwise. In addition, we used *Pearson*’s correlation analysis to investigate possible correlations between different metabolites and other variables related to renal function, cytokines, chemokines, inflammatory mediators, and the gut microbiota. Statistical analyses were performed in both directions, where *P* < 0.05 was considered significant.

## Results

### Phytochemical profiling and quantitative evaluation of the SSAEP

**Fig. 1:**
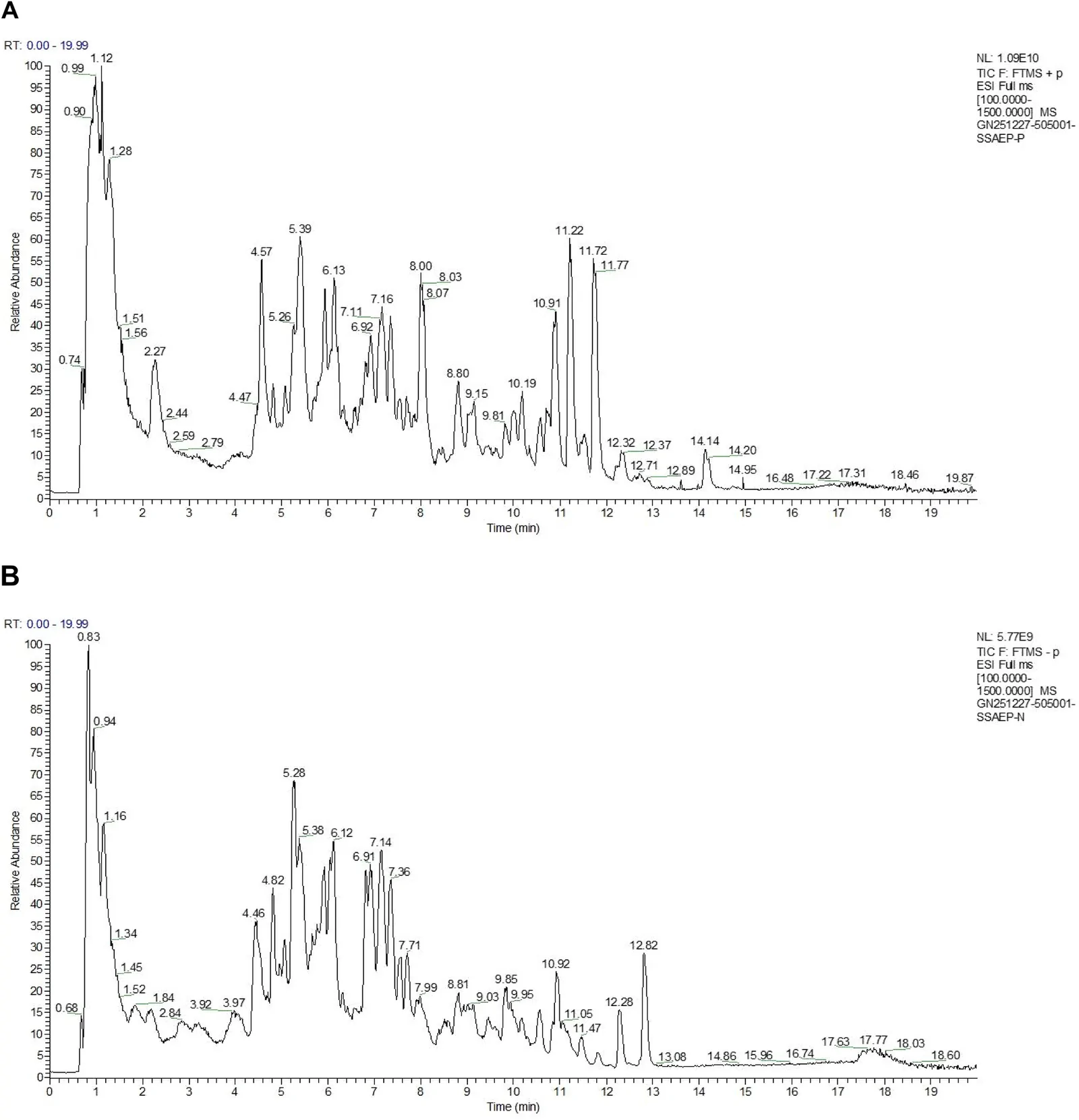
Total ion chromatograms (TICs) of the SSAEP in both positive and negative ionization modes. **A** TIC of the SSAEP obtained in positive ionization mode. **B** TIC of the SSAEP obtained in negative ionization mode

The chemical composition of the SSAEP was determined by ultrahigh-performance liquid chromatography‒tandem mass spectrometry (UHPLC‒MS/MS) in two different ionization modes (positive and negative) (Fig. 1A–B). Data analysis was performed on the basis of Compound Discoverer 3.3, which enables different analysis steps, such as retention time correction, peak detection or feature extraction. Compound identification was performed by comparing the second mass spectrum with the online Thermo mzCloud database as well as the local Thermo mzValut database. In addition, a systematic search of the relevant literature in various databases, such as the TCMSP (https://www.91tcmsp.com/), PubMed, and CNKI was conducted to screen reported phytochemicals in the eight herbs used for SSAEP. In this combined analysis, a total of 82 different compounds were identified (*Supplementary* *Table S1*). Most consisted of flavonoids, benzene and substituted derivatives, organooxygen compounds, carboxylic acids and derivatives, anthracenes, cinnamic acids and derivatives, isoflavonoids, prenol lipids, phenols, coumarins and derivatives, among others.

### SSAEP was very effective against kidney damage caused by CRF in rats

**Fig. 2:**
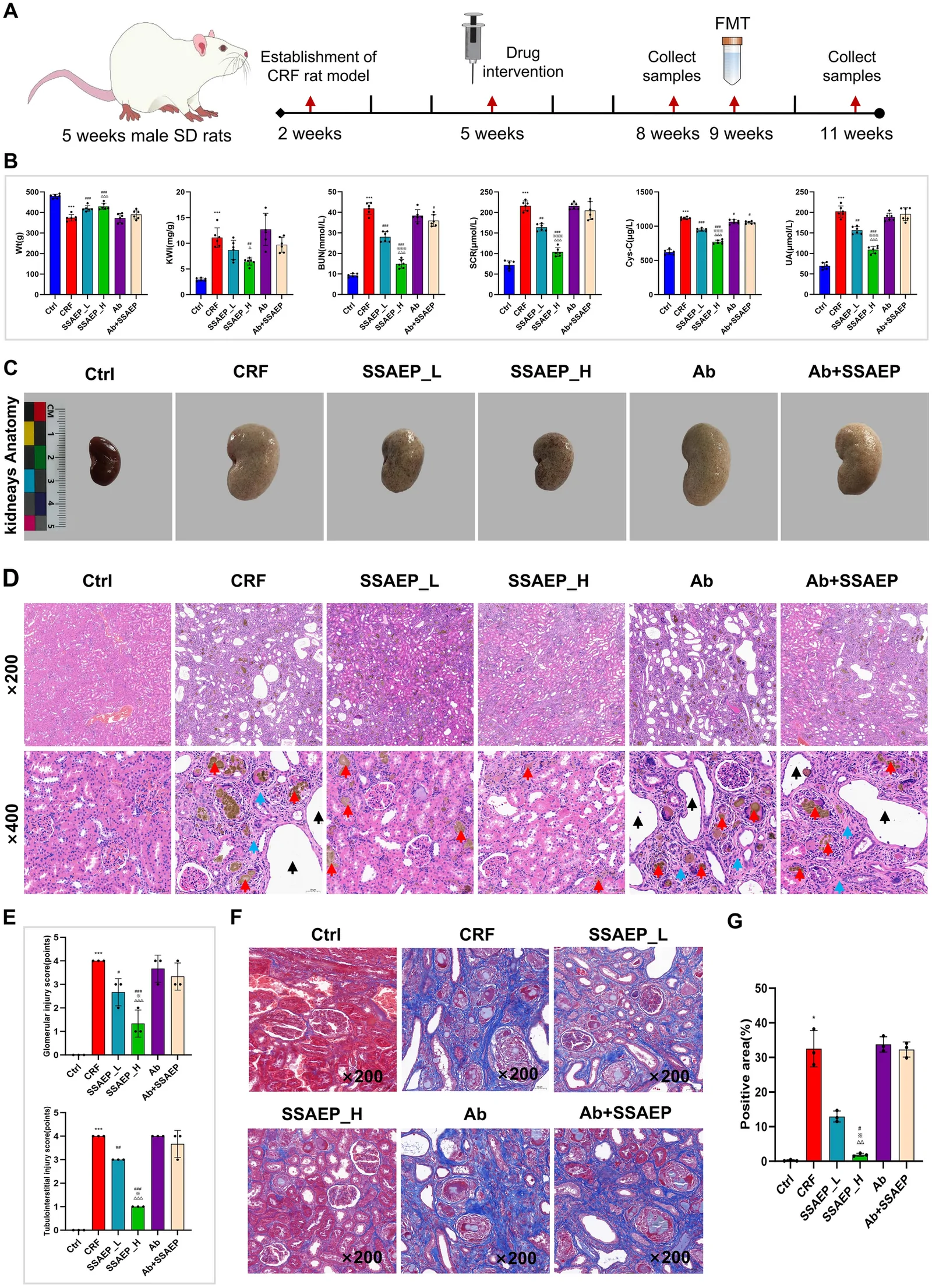
SSAEP ameliorates renal injury in a rat model of CRF. **A** Schematic representation of the experimental timeline for 5-week-old male SD rats. **B** Statistical analysis of the Wt, KWI, and renal function parameters (SUN, SCR, Cys-C, and UA) across the experimental groups. (n = 6. ^***^P < 0.001 vs. the Ctrl group; ^#^*P* < 0.05, ^##^*P* < 0.01, ^###^*P* < 0.001 vs. the CRF group; ^※※※^*P* < 0.001 vs. the SSAEP_L group; ^△△^*P* < 0.01, ^△△△^*P* < 0.001 vs. the Ab + SSAEP group). **C** Representative gross morphological appearance of kidneys from each group following a 4-week treatment period. **D** HE staining of renal tissue sections from each group (magnification: × 200 and × 400). **E** Glomerular injury score and tubulointerstitial injury score (n = 6). **F** Masson staining of renal tissue sections from each group (magnification: × 200). **G** Quantitative analysis of the percentage of collagen fiber area in Masson-stained renal sections from each group. (n = 3. ^*^P < 0.05 vs. the Ctrl group; ^#^*P* < 0.05 vs. the CRF group; ^※^*P* < 0.05 vs. the SSAEP_L group; ^△△^*P* < 0.01 vs. Ab + SSAEP group)

In total, there were six subject groups: Ctrl, CRF, SSAEP_L, SSAEP_H, Ab and Ab + SSAEP. The data revealed significant differences between the Ctrl group and all the treatment groups in terms of the Wt, renal mass index (KWI), and other kidney markers (SUN, SCR, Cys-C, and UA) (Fig. 2A–C). After four weeks of medication therapy, the KWI decreased significantly in the SSAEP_H group compared with that in the CRF group; moreover, compared with the SSAEP_H group, the SSAEP_L group experienced better weight gain and significant improvement in renal indices, suggesting that there was a dose–effect relationship between SSAEP treatment and clinical efficacy. In addition, compared with the Ab + SSAEP combined group, the SSAEP_H group showed greater improvement in all the indicators, and these differences were statistically significant (Fig. 2B).

Histopathological analysis revealed that the morphological structures of kidney tissues improved significantly after SSAEP treatment. HE staining revealed that kidney structure was almost restored to its original state after low- or high-dose SSAEP treatment, with only a few inflammatory cells observed (Fig. 2D). Semiquantitative scoring of renal pathology revealed that compared with the CRF group, SSAEP treatment, particularly at high doses, significantly reduced both the glomerular injury score and the tubulointerstitial injury score (Fig. 2E). Glomerular mesangial collagen was decreased in the high-dose SSAEP-treated group, as evidenced by the results of Masson staining (Fig. 2F). The quantification of collagen fibers differed significantly between the high-dose SSAEP group and the CRF group and between the high-dose SSAEP group and the Ab + SSAEP group (Fig. 2G).

Taken together, these findings indicate that SSAEP treatment positively impacts CRF-related renal dysfunction and that this effect is strongly associated with changes in the gut microbiota composition.

### Therapeutic effects of SSAEP on inflammatory responses and fibrotic changes in CRF rats

**Fig. 3:**
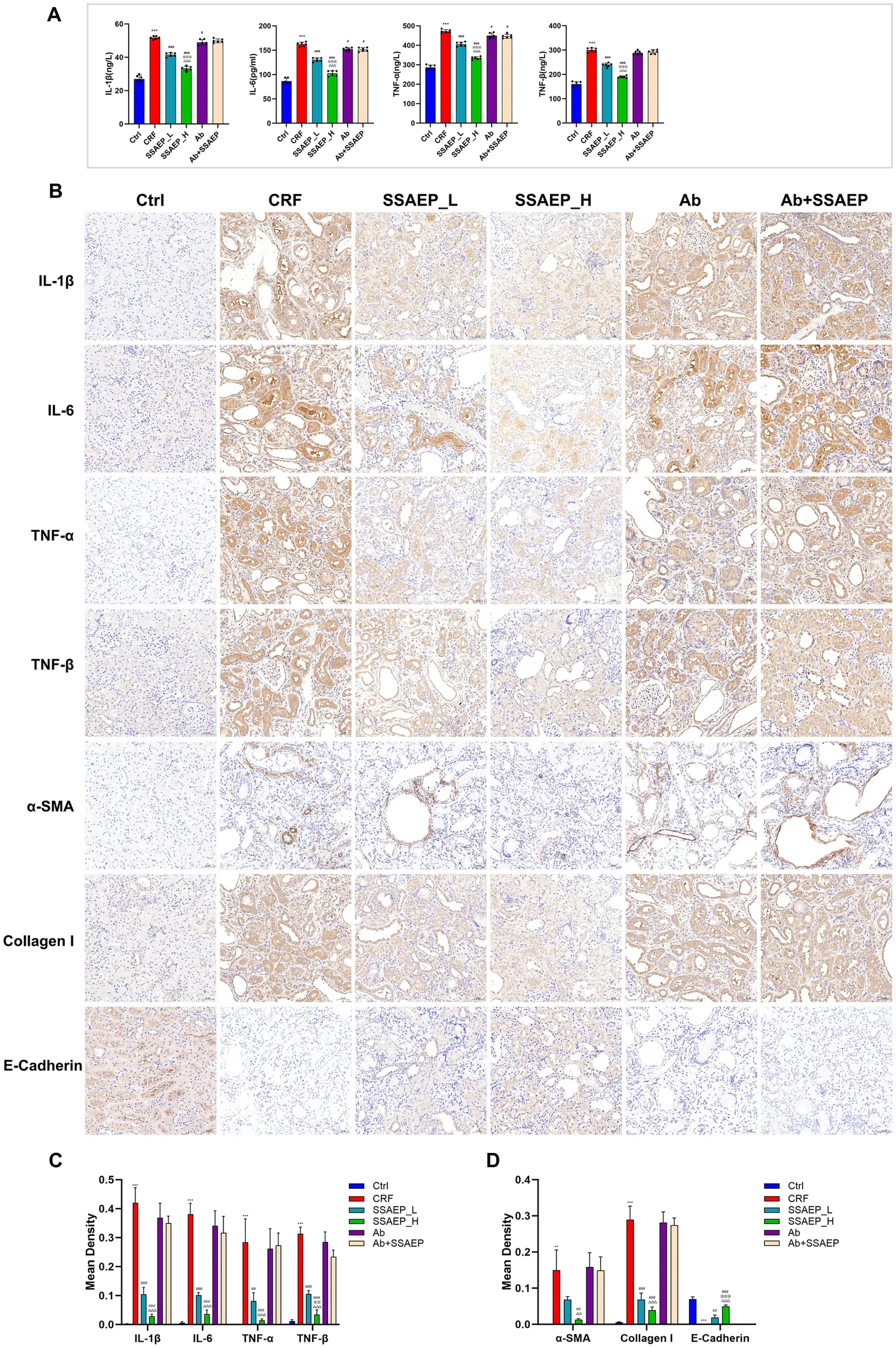
SSAEP attenuates inflammation and renal fibrosis in a rat model of CRF. **A** Quantitative analysis of serum inflammatory factor levels (IL-1β, IL-6, TNF-α, and TNF-β) across the experimental groups. (n = 6. ^***^P < 0.001 vs. the Ctrl group; ^###^*P* < 0.001 vs. the CRF group; ^※※※^*P* < 0.001 vs. the SSAEP_L group; ^△△△^*P* < 0.001 vs. Ab + SSAEP group.) **B** Representative immunohistochemical images illustrating the expression of inflammatory factors (IL-1β, IL-6, TNF-α, and TNF-β) and renal fibrosis markers (α-SMA, collagen I, and E-cadherin) in kidney tissues from each group. **C** Statistical evaluation of the mean density of inflammatory factors in renal tissues by IHC. (n = 3. ^***^P < 0.001 vs. the Ctrl group; ^##^*P* < 0. 01, ^###^*P* < 0.001 vs. the CRF group; ^※※^*P* < 0.01 vs. the SSAEP_L group; ^△△△^*P* < 0.001 vs. Ab + SSAEP group.) **D** Statistical assessment of the mean density of renal fibrosis markers determined by IHC. (n = 3. ^**^P < 0.01, ^***^P < 0.001 vs. the Ctrl group; ^##^*P* < 0.01, ^###^*P* < 0.001 vs. the CRF group; ^※※※^*P* < 0.001 vs. the SSAEP_L group; ^△△^*P* < 0.01, ^△△△^*P* < 0.001 vs. Ab + SSAEP group.)

The levels of inflammation and renal fibrosis biomarkers were measured in all six groups (the Ctrl, CRF, SSAEP_L, SSAEP_H, Ab, and Ab + SSAEP groups). ELISAs revealed the levels of several inflammatory factors, such as IL-1β, IL-6, TNF-α and TNF-β, in response to various treatments (Fig. 3A). After 28 days of pharmacologic intervention, the expression of each of these proinflammatory cytokines significantly decreased in response to both low- and high-dose SSAEP compared with that in response to CRF, with a greater effect at higher doses; furthermore, a comparison of the Ab + SSAEP treatment group versus the higher-dose SSAEP treatment group revealed that the latter had an additional significant decrease in inflammatory cytokine production.

The IHC results revealed differences in renal tissue expression between the different groups. The expression in the SSAEP_L group and the SSAEP_H group changed as follows: immunostaining for proinflammatory cytokines and profibrotic markers (α-SMA and collagen I) was significantly reduced. Moreover, the expression of E-cadherin was significantly increased in both groups (Fig. 3B), and further quantitative analysis confirmed these results, demonstrating that the average OD readings of IL-1β, IL-6, TNF-α and TNF-β in kidney tissue obtained from SSAEP-administered rats were significantly lower than those in kidney tissue from rats treated with CRF alone, with the reductions being statistically significant in all cases (Fig. 3C). Compared with those in the Ab + SSAEP group, the OD values of inflammatory factors decreased significantly in the SSAEP_H group (Fig. 3C). With respect to fibrosis indicators, kidney staining revealed decreased α-SMA and collagen I but increased E-cadherin in the SSAEP_H group (Fig. 3B). Compared with those in the CRF group, the optical densities of α-SMA and collagen I in the SSAEP_H group were significantly lower, but the levels of E-cadherin were significantly greater (Fig. 3D); moreover, compared with those in the Ab + SSAEP group, the OD of the SSAEP_H group continued to significantly increase, as shown by the significantly increased E-cadherin OD (Fig. 3D).

In conclusion, these results suggest that the ability of the SSAEP to reduce inflammation and fibrosis is dependent on an intact gut microbiota, as evidenced by the results of Ab and FMT experiments.

### SSAEP reduces inflammation and fibrosis in the kidneys of CRF rats by regulating the gut microbiota

**Fig. 4:**
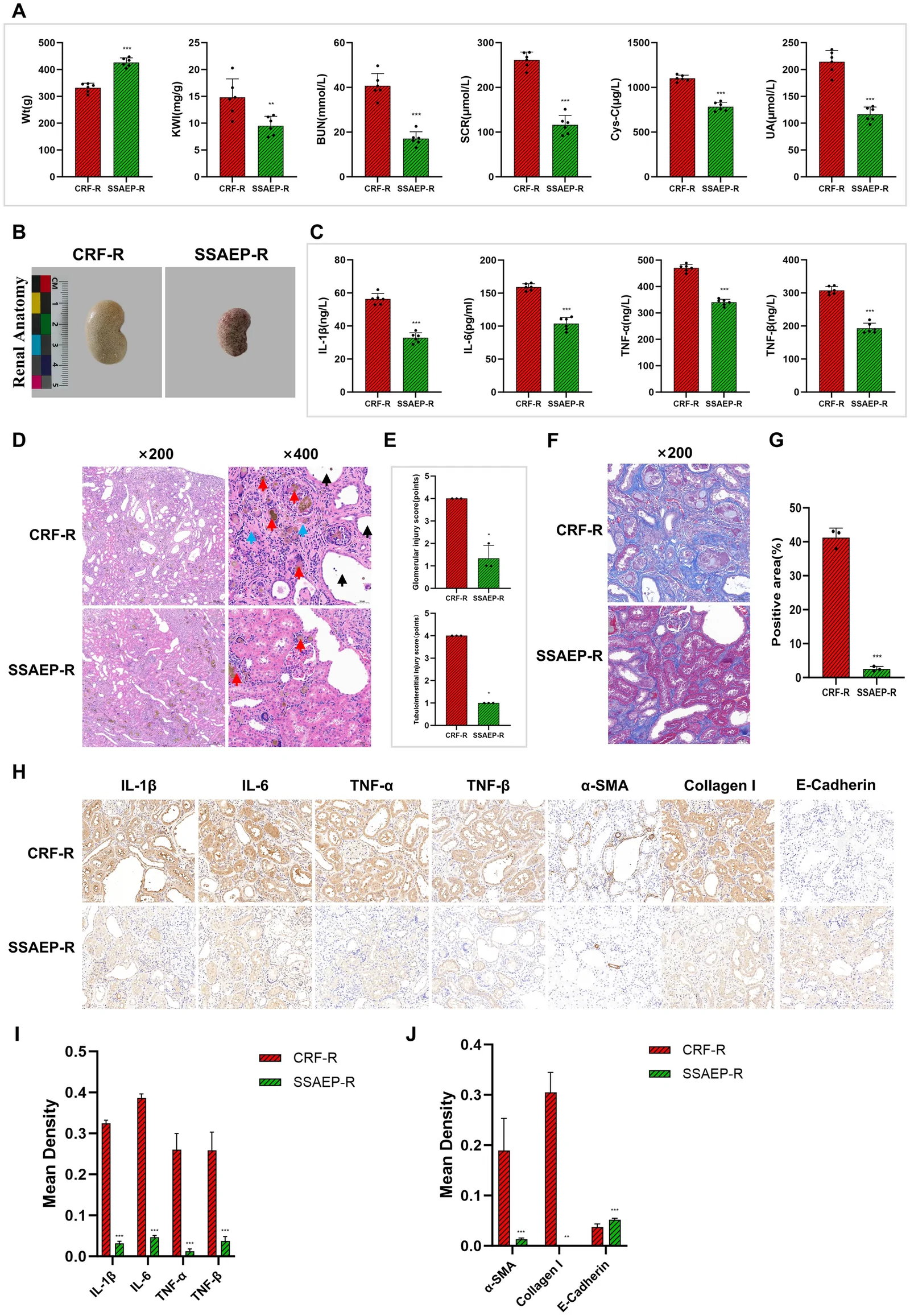
SSAEP ameliorates inflammation and renal fibrosis in CRF rats via modulation of the gut microbiota. **A** Statistical analysis of the Wt, KWI, and renal function parameters (SUN, SCR, Cys-C, UA) across the experimental groups. (n = 6. ^**^P < 0.01, ^***^P < 0.001 vs. the CRF-R group). **B** Representative gross anatomical and morphological images of kidneys harvested from each group following a 2-week FMT protocol. **C** Quantitative analysis of the levels of inflammatory factors (IL-1β, IL-6, TNF-α, and TNF-β) in serum samples from all the groups. (n = 6. ^***^P < 0.001 vs. the CRF-R group). **D** Histopathological evaluation of renal tissue by HE staining (magnification: × 200 and × 400). **E** Glomerular injury score and tubulointerstitial injury score (n = 6). **F** Assessment of renal collagen deposition via Masson staining (magnification: × 200). **G** Quantification of the percentage of collagen fibers in Masson-stained kidney sections. (n = 3. ^***^P < 0.001 vs. the CRF-R group). **H** IHC staining demonstrating the expression and localization of key inflammatory factors (IL-1β, IL-6, TNF-α, and TNF-β) and renal fibrosis markers (α-SMA, collagen I, and E-cadherin) in renal tissues. **I** Statistical analysis of the mean density for IHC staining of inflammatory factors (IL-1β, IL-6, TNF-α, and TNF-β) in kidney sections. (n = 3. ^***^P < 0. 001 vs. the CRF-R group). **J** Statistical analysis of the mean density of kidney fibrosis markers (α-SMA, collagen I, and E-cadherin) by IHC staining. (n = 3. ^**^P < 0.01, ^***^P < 0.001 vs. the CRF-R group)

These results suggest that treatment with SSAEP can protect against kidney injury caused by CRF in rats via anti-inflammatory effects and reduced renal fibrosis, which may be associated with changes in the composition and structure of the gut flora. To validate the hypothesis above and establish a causal role of the gut microbiota as an intermediate, we collected stools from rats in the CRF group and SSAEP_H group, which had been previously used for FMT experiments, and then prepared stool suspension solutions and delivered them into rats in the CRF-R group or SSAEP_R group accordingly. After the administration of the drugs for 14 days, pathological changes in the kidney were observed, and remarkable improvement was observed after drug intervention. Quantitative evaluation revealed that compared with the CRF-R control group, the SSAEP-R group had significant increases in the Wt and decreases in the KWI and significant increases in various indicators of kidney function, such as BUN, SCR, Cys-C, and UA levels *(*Fig. 4A–B). ELISAs also confirmed that the levels of proinflammatory cytokines (IL-1β, IL-6, TNF-α and TNF-β) were significantly reduced in the SSAEP-R group. The experimental results demonstrated that the values in the SSAEP-R group were considerably lower than those observed in the CRF-R group, indicating statistically significant differences (Fig. 4C).

Compared with those in the CRF-R group, the pathological indicators in renal tissues in the SSAEP-R group were significantly improved (Fig. 4D). Semiquantitative scoring of renal pathology revealed that compared with the CRF-R group, the SSAEP-R treatment group had significantly lower glomerular injury scores and tubulointerstitial injury scores (Fig. 4E). Masson staining revealed that the deposition of collagen fibers in glomerular mesangial areas was reduced in the SSAEP-R group (Fig. 4F). Quantification of the proportion of surface area covered by collagen fibers revealed that this feature was significantly lower for SSAEP-R than for CRF-R (Fig. 4G).

The results of the IHC staining showed that the protein expression significantly differed among the three groups. In kidney tissue samples from the SSAEP-R group, immunostaining for proinflammatory (IL-1β, IL-6, TNF-α, and TNF-β) and profibrotic (a-SMA and collagen I) markers was significantly reduced. Conversely, the expression of E-cadherin increased with increasing staining intensity (Fig. 4H). Densitometry revealed that compared with the CRF-R controls, the SSAEP-R animals had significantly lower average OD values for each of these tested inflammatory mediators (Fig. 4I), as well as for extracellular matrix components, with decreased α-SMA and collagen I expression and elevated E-cadherin expression in the treatment group. These changes were statistically significant (Fig. 4J).

Finally, FMT experiments provide causal evidence that the therapeutic benefits of SSAEP are transferable via the gut microbiota, strongly supporting a microbiome-dependent mechanism.

### SSAEP improves the ecological balance of the gut microbiota in CRF rats

To explore the mechanism through which SSAEP treatment enhances kidney function in CRF rats via intestinal flora, stool samples were collected and prepared for 16S rDNA sequencing; variable regions of the 16S rRNA gene, V3 and V4, were amplified by polymerase chain reaction (PCR) and sequenced. Following simple preprocessing steps such as demultiplexing, assembly, and quality filtering, we obtained high-quality sequences for further analyses. Taxonomic assignment of microbes was based on the operational taxonomic unit (OTU) clustering approach.

**Fig. 5:**
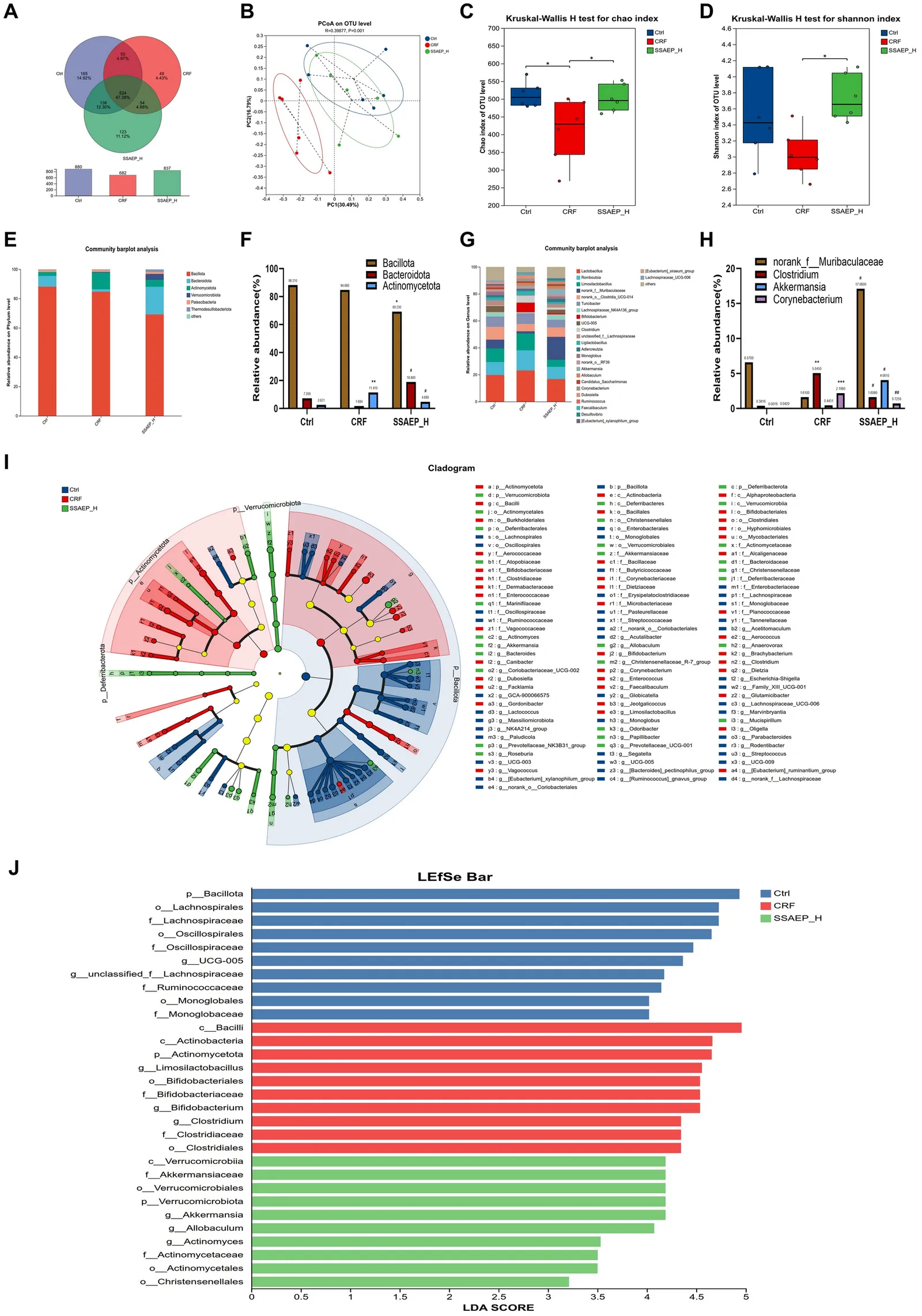
SSAEP enhances the ecological equilibrium of the gut microbiota in rats with CRF (n = 6). **A** Venn diagram illustrating the overlap of OTU sequences among the Ctrl, CRF, and SSAEP_H groups. **B** PCoA depicting the microbial community structure for each experimental group. **C** Chao1 index analysis revealed significant differences in microbial community richness between groups (^*^P < 0.05). **D** Shannon index analysis demonstrated notable variations in microbial diversity across groups (^*^P < 0. 05). **E** Bar chart representing the relative abundance of the gut microbiota at the phylum level in each rat group. **F** Ratios of the relative abundance of dominant bacterial phyla in the intestinal tracts of rats across different groups (^*^P < 0.05). **G** Bar chart showing the relative abundance of the gut microbiota at the genus level in each group of rats. **H** Ratios of the relative abundance of the dominant bacterial genera within the intestinal microbiota of the rats in each group (.^*^P < 0.05). **I** Multilevel phylogenetic tree generated by LEfSe analysis for the gut microbiota across different groups of rats. **J** LDA discriminant bar plots for the gut microbiota in each rat group (higher LDA scores indicate a greater contribution of species abundance to the observed differential effects)

Using Venn diagram plots, we detected a total of 1,106 unique OTUs in each treatment group, with 1,106 different types of intestinal flora. Among them, 524 OTUs were common to the 3 groups (Fig. 5A). The PCoA showed an obvious clustering trend (Fig. 5B), where the data in each treatment group were clustered together but separated from the others. The results revealed clear spatial segregation of the control and CRF groups on the PCoA plot; however, the SSAEP_H group fell in the middle with greater proximity to the control group, implying shifts in microbiota composition (measured via beta diversity measures). SSAEP_H partially recovered the reduced intestinal microflora beta diversity in CRF rats. The diversity of each microbiome was analyzed in terms of the number of species or richness by calculating the Chao index (Fig. 5C) and as a measure of the evenness with which these species are distributed within each microbiome using the Shannon index (Fig. 5D). The results revealed that the alpha diversity parameters were significantly lower in the CRF group and that SSAEP_H reversed these changes to a great extent.

At the phylum level, we also analyzed the differences in composition between the groups (Fig. 5E–F) and found that compared with that in the other two groups, the abundance of *Bacteriodota* was significantly greater but that the abundance of *Actinomycetota* was lower after treatment with SSAEP_H (Fig. 5B). At the genus level, we also analyzed the differences in composition among the three groups (Fig. 5G–H) and found that after treatment with SSAEP_H, the abundance of *Clostridium* and *Corynebacterium* clearly decreased. This was accompanied by dramatic increases in *norank_f__Muribaculaceae* and *Akkermansia*. To identify the bacterial species whose abundance differed between groups, we performed LEfSe (Fig. 5I–J), which revealed that *Actinomycetota*, *Bifidobacterium*, *Clostridium*, *Clostridiaceae*, and *Clostridiales* were specific to the microbiota of the CRF group, whereas *Verrucomicrobiia*, *Akkermansia*ceae, *Akkermansia* and *Allobaculum* were the dominant genera in the SSAEP_H treatment group.

### Impacts of SSAEP on the metabolic profiles of the serum and colon contents of CRF rats

To assess potential changes in metabolites, which serve as crucial intermediate substances linking changes in the gut microbiota to host physiology, a detailed metabolomics analysis was carried out using serum and colon content samples from Ctrl, CRF, and SSAEP_H group rats. Using a step-by-step screening method, we were detected and characterized potentially relevant variations in metabolite expression profiles.

**Fig. 6:**
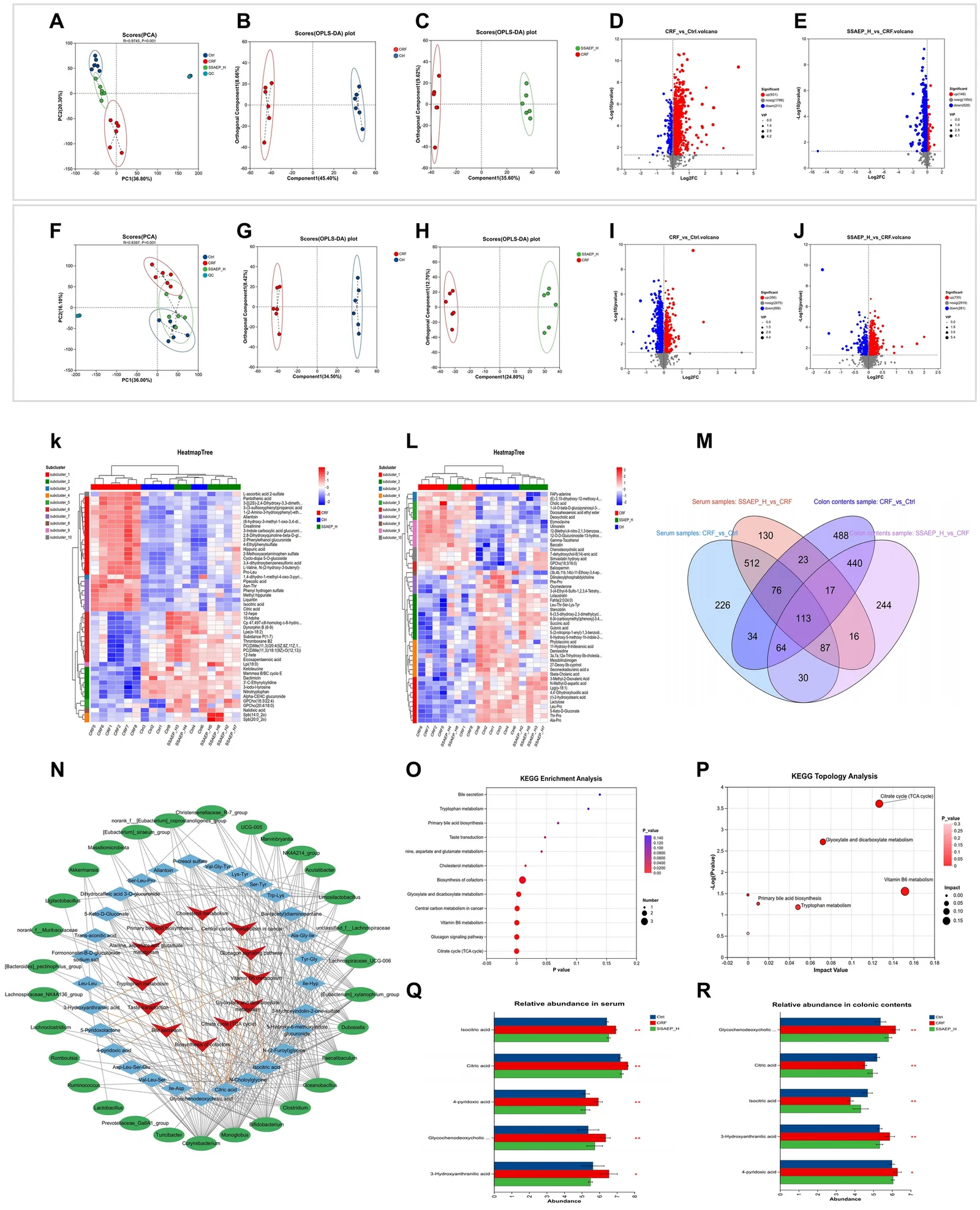
Impacts of the SSAEP on the metabolite profiles in the serum and colonic contents of CRF rats (n = 6). **A** Serum samples: PCA results for the three groups. **B** Serum samples: OPLS-DA comparing the CRF group with the Ctrl group. **C** Serum samples: OPLS-DA results comparing the SSAEP_H group with the CRF group. **D** Serum samples: Volcano plot illustrating differentially abundant metabolites between the CRF and Ctrl groups. **E** Serum samples: Volcano plot showing differentially abundant metabolites between the SSAEP_H and CRF groups. **F** Colonic content samples: PCA results for the three groups. **G** Colonic content samples: OPLS-DA comparing the CRF group with the Ctrl group. **H** Colonic content samples: OPLS-DA results comparing the SSAEP_H group with the CRF group. **I** Colonic content samples: Volcano plot of differentially abundant metabolites between the CRF and Ctrl groups. **J** Colonic content samples: Volcano plot of differentially abundant metabolites between the SSAEP_H and CRF groups. **K** Serum samples: Heatmap depicting clustering of differentially abundant metabolites across groups. **L** Colonic content samples: Heatmap showing metabolite clustering among different groups. **M** Venn diagram representing overlapping differentially abundant metabolites in the serum and colonic content samples. **N** The “gut microbiota–metabolite–pathway” network. **O** KEGG enrichment analysis revealed 12 metabolic pathways significantly enriched by 29 differentially abundant metabolites. **P** KEGG topology analysis of the 29 differentially abundant metabolites revealed 7 significantly enriched metabolic pathways and 5 key differentially abundant metabolites. **Q** Statistical analysis of differences in the relative abundance of the 5 differentially abundant metabolites in serum samples (^*^P < 0.05, ^**^P < 0.01). **R** Statistical analysis of differences in the relative abundance of the 5 differentially abundant metabolites in the colonic content samples (^*^P < 0.05, ^**^P < 0.01)

In our first analyses, we sought to characterize the metabolic profiles of the serum and colonic contents for each cohort of rats in all ionization modes. For the serum metabolomics analysis, PCA was first applied to the serum metabolite dataset, yielding a model with *R* = *0.9745* and *P* = *0.001* (*PC1* = *36.80%, PC2* = *20.30%*), indicating robust separation among the Ctrl, CRF, and SSAEP_H groups (Fig. 6A). Subsequent multivariate statistics via OPLS-DA further confirmed clear discrimination between the CRF cohort and both the Ctrl and SSAEP_H-treated groups *(*Fig. 6B–C). Differentially abundant metabolite analysis (*VIP* > *1, FC* > *1, P* < *0.05*) revealed that comparing serum metabolites from the CRF group with those from the control group yielded 1,042 significantly changed metabolites (Fig. 6D–E), whereas 974 metabolites were significantly different between the SSAEP_H and CRF groups. Similarly, PCA of the colonic content metabolome demonstrated clear separation, with *R* = *0.8357, P* = *0.001* (*PC1* = *36.00%, PC2* = *16.10%*) (Fig. 6F). The same trend was observed in the OPLS-DA models (Fig. 6G–H). A comparison of the metabolite profiles of the colonic contents (Fig. 6I–J) revealed 1,255 metabolites that were significantly differentially expressed between the CRF and Ctrl groups, whereas 1,011 metabolites were significantly different between the SSAEP_H and CRF groups. In summary, both the PCA and OPLS-DA results consistently highlighted pronounced metabolic perturbations induced by CRF, which were partially reversed by SSAEP_H treatment. Compared with the serum samples, the colonic content samples presented a greater number of differentially abundant metabolites, suggesting more extensive metabolic remodeling at the gut level. A cluster heatmap (Fig. 6K–L) revealed that the abundance of metabolites whose abundance was higher or lower in the CRF group was lower or higher, respectively, in the SSAEP_H group, indicating the modulation of specific pathways under each condition.

To identify key differentially expressed metabolites accurately, we used a multi-step filtering strategy that included the following: (1) comparison of the differentially abundant metabolites in the serum and colon contents between the two treatment groups, identification of 113 expressed metabolites using a Venn diagram (*Supplementary Table S2*); (2) selection of the up- or downregulated metabolites identified for “CRF vs. Control”, which showed opposite trends in their expression for “SSAEP_H vs. CRF”; and (3) filtering of this list using a combination of prior knowledge and querying in databases, retaining only those that are produced by gut microbes. After this iterative process, we ultimately identified 29 key differentially expressed metabolites from the 113 common metabolites (Table 3).

**Table 3:** The final identification of 29 differentially abundant metabolites was accomplished through a series of rigorous screening procedures

No	Differentially abundant metabolite	Metabolite type	Serum samples			Colonic contents samples			Microbial community-associated metabolites (yes or no)
			VIP	CRF_vs_Ctrl	SSAEP_H_vs_CRF	VIP	CRF_vs_Ctrl	SSAEP_H_vs_CRF	
1	Val-Leu-Ser	Tripeptide	3.4080	up	down	2.2355	down	up	yes
2	Ile-Hyp*	Dipeptide	3.0107	up	down	1.0641	up	down	yes
3	Lys-Tyr	Dipeptide	2.7274	up	down	2.0188	down	up	yes
4	Ser-Leu-Pro	Tripeptide	2.6802	up	down	1.5323	down	up	yes
5	Trp-Lys	Dipeptide	2.4175	up	down	1.2330	down	up	yes
6	3-Hydroxyindolin-2-one-sulfate	Sulfated oxindole derivative	2.3389	up	down	2.2217	down	up	yes
7	3-Hydroxyanthranilic acid*	Hydroxy anthranilic acid derivative	2.3214	up	down	1.9578	up	down	yes
8	Asp-Leu-Ser-Glu*	Tetrapeptide	2.1698	up	down	2.1397	up	down	yes
9	Ser-Tyr	Dipeptide	2.1445	up	down	1.6979	down	up	yes
10	5-Hydroxy-6-methoxyindole glucuronide	Hydroxy methoxy indole glucuronide	2.0776	up	down	1.5532	down	up	yes
11	4-pyridoxic acid*	Pyridoxine metabolite	1.9437	up	down	1.1769	up	down	yes
12	Val-Gly-Tyr	Tripeptide	1.8891	up	down	1.6891	down	up	yes
13	Bis-(acetyl)diaminopentane*	Acetylated polyamine	1.8627	up	down	2.1575	up	down	yes
14	Dihydrocaffeic acid 3-O-glucuronide	Hydroxycinnamic acid glucuronide	1.8381	up	down	1.9081	down	up	yes
15	Leu-Leu*	Dipeptide	1.8107	up	down	2.3354	up	down	yes
16	P-cresol sulfate*	Sulfated phenol derivative	1.7346	up	down	1.0687	up	down	yes
17	Tyr-Gly	Dipeptide	1.6687	up	down	1.4569	down	up	yes
18	Glycochenodeoxycholic acid*	Bile acid conjugate	1.5952	up	down	1.5957	up	down	yes
19	N-Choloylglycine*	Bile acid conjugate	1.5287	up	down	1.3656	up	down	yes
20	Isocitric acid	Tricarboxylic acid cycle intermediate	1.5286	up	down	1.8425	down	up	yes
21	Formononetin-B-D-glucuronide sodium salt	Isoflavone glucuronide conjugate	1.4966	up	down	1.663	down	up	yes
22	5-Pyridoxolactone*	Pyridoxine derivative	1.4675	up	down	1.2481	up	down	yes
23	Citric acid	Tricarboxylic acid cycle intermediate	1.4157	up	down	1.6634	down	up	yes
24	Ala-Gly-Ile	Tripeptide	1.2794	up	down	1.3029	down	up	yes
25	Ile-Asp	Dipeptide	1.259	up	down	1.7741	down	up	yes
26	Trans-aconitic acid	Tricarboxylic acid	1.1600	up	down	2.5354	down	up	yes
27	Allantoin*	Purine metabolite	1.1114	up	down	1.9700	up	down	yes
28	N-(2-Furoyl)glycine	Furoyl amino acid conjugate	1.0759	up	down	1.8472	down	up	yes
29	5-Keto-D-Gluconate	Keto gluconate derivative	1.0420	up	down	1.6187	down	up	yes

The table above is sorted according to the VIP values in the screened serum samples; * indicates that the upregulation and downregulation trends are consistent between the serum samples and the colonic contents in each group; up represents upregulation, and down represents downregulation

Next, we carried out a more detailed analysis of the 29 differentially expressed metabolites. To further elucidate the causal cascade linking altered gut microbiota to host metabolic and signaling pathways, we identified significant correlations between the expression of the 29 key differentially abundant metabolites and the abundance of the genus-level microbial taxa that were differentially abundant among the Ctrl, CRF, and SSAEP_H groups. The identified metabolite–microbe associations were then mapped to the KEGG pathways enriched by these metabolites. Using Cytoscape (v3.10.2), we constructed an integrated “gut microbiota–metabolite–pathway” network (Fig. 6N). Analysis of the KEGG pathways enriched by these metabolites revealed 12 putatively relevant metabolic pathways (Fig. 6O). A subsequent fine-grained topological analysis of these pathways revealed that seven specific pathways, notably vitamin B6 metabolism and the citric acid cycle (TCA cycle), were significantly enriched (Fig. 6P). Comparative analysis revealed significant differences in the levels of five metabolites, namely, 4-pyridoxic acid, isocitric acid, citric acid, glycochenodeoxycholic acid, and 5-pyridoxolactone. Detailed information regarding the aforementioned seven major metabolic pathways and five core metabolites is presented in Table 4.

**Table 4:** Statistical analysis of 7 major metabolic pathways and 5 key metabolites identified through KEGG topology analysis

No	Metabolite pathway	Impact	*P*	Metabolite(s)
1	Vitamin B6 metabolism	0.152	0.028	4-pyridoxic acid
2	Citrate cycle (TCA cycle)	0.127	0.000	Isocitric acid; citric acid
3	Glyoxylate and dicarboxylate metabolism	0.072	0.002	Isocitric acid; citric acid
4	Tryptophan metabolism	0.048	0.067	3-Hydroxyanthranilic acid
5	Primary bile acid biosynthesis	0.010	0.055	Glycochenodeoxycholic acid
6	Biosynthesis of cofactors	0.000	0.276	3-Hydroxyanthranilic acid
7	Alanine, aspartate and glutamate metabolism	0.000	0.034	Citric acid

The relative levels of the top five DEMs in each group were statistically compared (Fig. 6Q–R), and the results revealed significant differences. The levels of 4-pyridoxic acid, 3-hydroxyanthranilic acid and glycochenodeoxycholic acid in the CRF group were greater than those in serum samples, whereas the levels of these metabolites were significantly lower in the SSAEP_H-treated group. The same trend was observed for the colon content samples.

After a rigorous series of filtering steps, we identified 4-pyridoxic acid, 3-hydroxyanthranilic acid and glycochenodeoxycholic acid as significantly expressed metabolites that are likely affected by SSAEP administration.

### Metabolic pathway of GCDCA and its interplay with the gut microbiome

To elucidate the mechanistic association between the gut microbiota and the pivotal metabolite GCDCA, we delineated its specific biosynthetic pathway (*Supplementary Fig S1*). GCDCA, a secondary bile acid, is primarily generated in the intestinal lumen through the deconjugation and subsequent 7α-dehydroxylation of primary bile acids such as glycocholic acid (GCA), a process mediated by specific bacterial taxa, notably *Clostridium* genus. In the CRF model, a marked expansion of *Clostridium* accelerated the conversion of GCA to GCDCA, leading to its systemic accumulation. Conversely, SSAEP intervention significantly suppressed *Clostridium* abundance, thereby attenuating this metabolic flux and reducing GCDCA levels.

Concurrently, SSAEP treatment significantly increased the abundance of *Akkermansia*, a genus known for mucin degradation and maintenance of gut barrier integrity. The relative abundance of *Akkermansia* was negatively correlated with GCDCA levels, suggesting an indirect mechanism whereby *Akkermansia* contributes to GCDCA reduction by restoring gut ecological balance. Specifically, *Akkermansia* promotes the growth of butyrate-producing bacteria and reinforces the intestinal barrier, thereby reducing bacterial translocation and dampening the pro-inflammatory milieu that drives dysbiosis. This ecological restoration indirectly suppresses the metabolic activity of *Clostridium* and other GCDCA-producing bacteria, further lowering GCDCA concentrations. These findings establish a clear causal chain: *Clostridium* overgrowth-driven dysbiosis leads to aberrant GCDCA accumulation, while *Akkermansia* restoration counteracts this process by reshaping the gut microenvironment, ultimately influencing the pathological progression of CRF.

### Analysis of the relationships among differentially abundant metabolites, gut microbiota and related biomarkers

**Fig. 7:**
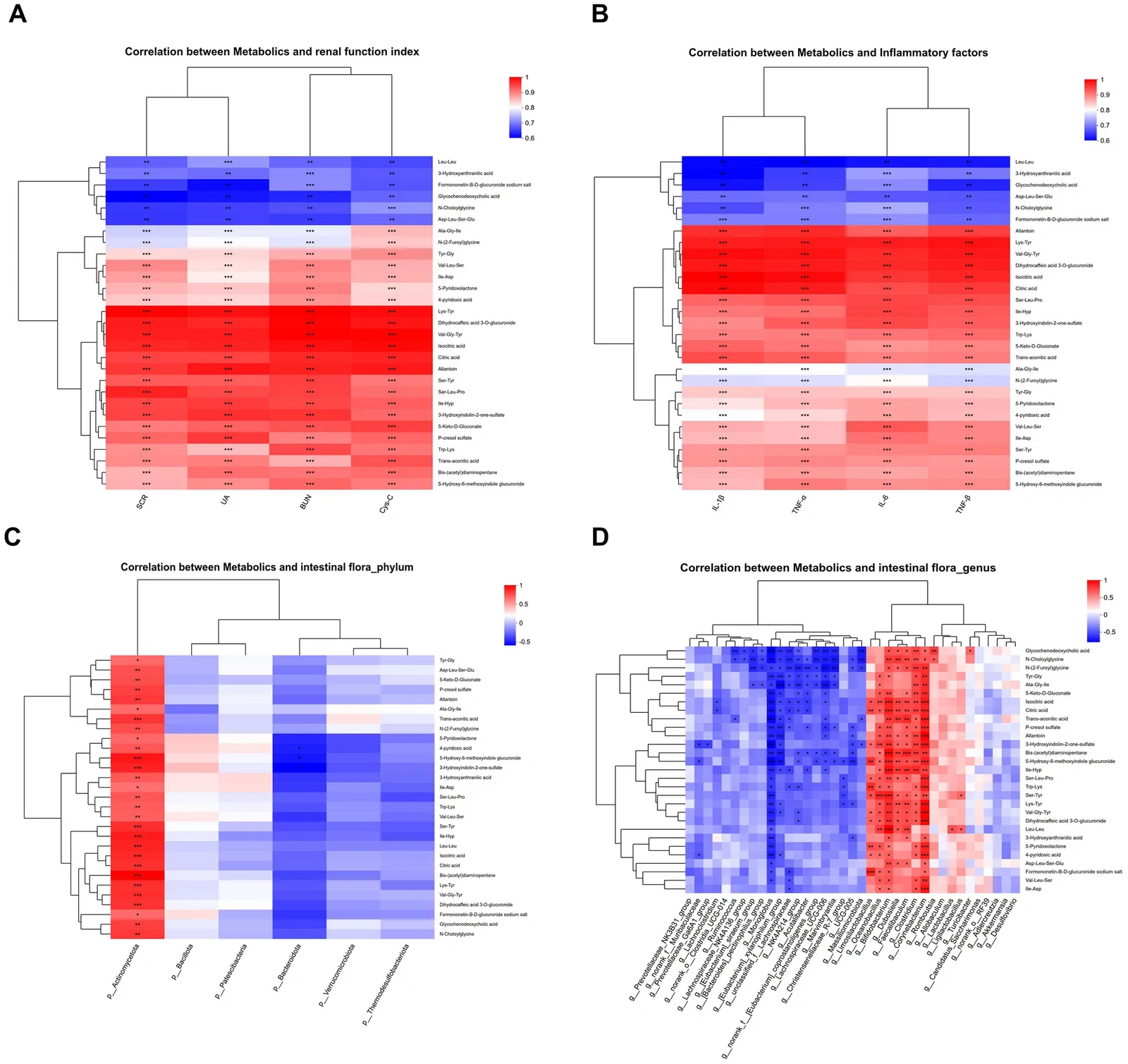
Analysis of the associations between differentially abundant metabolites and related indicators (^*^P < 0.05, ^**^P < 0.01, ^***^P < 0.001). **A** Correlation analysis between differentially abundant metabolites and renal function indicators. **B** Correlation analysis between differentially abundant metabolites and inflammatory factors. **C** Correlations between differentially abundant metabolites and the gut microbiota at the phylum level. **D** Correlation analysis between differentially abundant metabolites and genus-level differences in the gut microbiota

*Pearson*’s correlation analysis was used in this study to explore the correlations between different metabolites and other physiological indices,. Such as renal biomarkers, inflammation indicators, and the gut microbiota profile among the groups. The correlation map cutoff was set to a *P value* < *0.05* and *|r|*> *0.5*. The results revealed significant correlations between the top three candidate biomarkers, pyridoxic acid, 3-hydroxyanthranilic acid and glycochenodeoxycholic acid, and BUN, SCR, Cys-C and UA (Fig. 7A). The same compounds were consistently positively related to proinflammatory cytokines (IL-1β, IL-6, TNF-α, and TNF-β) (Fig. 7B). To explore the associations between these key metabolites and the gut microbiota, we first performed correlation analysis at the phylum level (Fig. 7C). This analysis revealed a broad trend: the three metabolites were positively correlated with the *Actinomycetota* phylum, whereas 4-pyridoxic acid was negatively correlated with the *Bacteroidota* phylum. While this provides a high-level overview, we recognize that the phylum-level resolution is coarse. To obtain more refined biological insight, we further conducted a detailed correlation analysis at the genus level (Fig. 7D), which revealed specific bacterial genera that drove the observed phylum-level trends. For instance, consistent with the phylum-level results, we found that the three metabolites were positively correlated with the relative abundance of the genera *Clostridium* and *Corynebacterium* but strongly negatively correlated with the relative abundance of *Akkermansia*. These genus-level findings substantiate our conclusions and highlight specific microbial players, such as *Akkermansia* and *Clostridium*, as the likely mediators of host–microbe cometabolism influencing CRF.

These findings suggest that 4-pyridoxic acid, 3-hydroxyanthranilic acid and glycochenodeoxycholic acid are positively correlated with not only indicators of renal failure and global inflammation but also etiological microbiota communities that are associated with CRF, while these compounds have poor bioavailability or are poorly absorbed and thus interact adversely with bacteria with potential beneficial effects. These metabolites represent potential intermediate substances involved in the indirect action of SSAEP, bridging the altered gut microbiota with the observed systemic and renal pathological changes.

### Network pharmacology and molecular docking-based preliminary exploration of SSAEP targets in the suppression of CRF

**Fig. 8:**
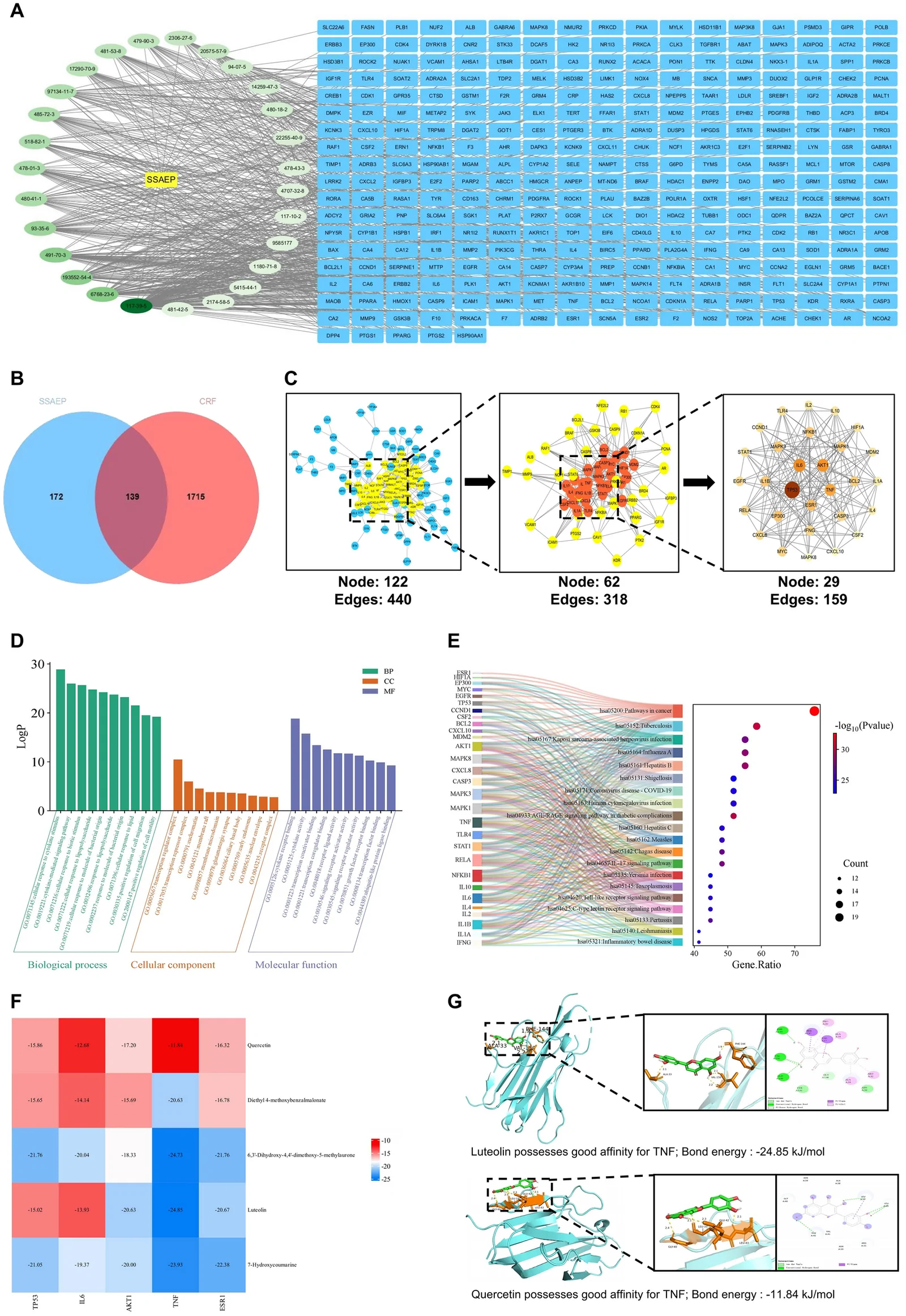
Preliminary investigation of the targets of the SSAEP in the suppression of CRF based on network pharmacology and molecular docking. **A** The “SSAEP active ingredient–target" network diagram comprising 27 compounds and 311 targets. **B** Venn diagram illustrating 139 overlapping targets between SSAEP and CRF. **C** PPI network and the final selection of 29 core targets. **D** GO analysis of the 29 core targets. **E** KEGG pathway enrichment analysis of the 29 core targets. **F** Heatmap displaying molecular docking results for five core compounds of the SSAEP against five core target proteins. (The numerical values in the figure indicate binding energy, expressed in kJ/mol.) **G** Schematic representation of the interaction between luteolin and TNF-encoded proteins

To screen potential pharmacological components and their pharmacological mechanism in response to SSAEP in CRF rats, UHPLC‒MS/MS technology was used for preliminary screening of the SSAEP sample composition, and a total of 82 effective chemical components were obtained. We subsequently used the traditional Chinese medicine systems pharmacology (TCMSP) and Swiss Target Prediction databases for target identification and prediction of the above molecules, leading to a total of 27 major active compounds (Table 5) and prediction of 311 possible target molecules. An interactive network map between the bioactive compound(s) present in SSAEP and its respective targets was constructed and visualized using Cytoscape software v3.10.2 (Fig. 8A).

**Table 5:** Information on 27 key active compounds identified in the SSAEP

No	Identity	Molecular formula	CAS/PubChem CID	Degree	Caco-2 in TCMSP	DL in TCMSP	Gl absorption In SwissADME	DL in SwissADME
1	Quercetin	C15H10O7	117-39-5	124	0.04	0.27	-	-
2	Diethyl 4-methoxybenzalmalonate	C15H18O5	6768-23-6	52	-	-	High	5"Yes"
3	6, 3'-Dihydroxy-4, 4'-dimethoxy-5-methylaurone	C18H16O6	193,552-54-4	49	-	-	High	5"Yes"
4	Luteolin	C15H10O6	491-70-3	49	0.18	0.24	-	-
5	7-Hydroxycoumarine	C9H6O3	93-35-6	41	-	-	High	3"Yes"and 2"No"
6	Naringenin	C15H12O5	480-41-1	32	0.28	0.21	-	-
7	Nobiletin	C21H22O8	478-01-3	31	1.05	0.51	-	-
8	Emodin	C15H10O5	518-82-1	31	0.22	0.23	-	-
9	Formononetin	C16H12O4	485-72-3	30	0.78	0.21		
10	(−)-pinellic acid	C18H34O5	97,134-11-7	29	-	-	High	4"Yes"and 1"No"
11	Isosinensetin	C20H20O7	17,290-70-9	23	1.15	0.44	-	-
12	Tangeretin	C20H20O7	481–53-8	23	1.23	0.42	-	-
13	Artemetin	C20H20O8	479–90-3	20	0.81	0.47	-	-
14	Sinensetin	C20H20O7	2306–27-6	19	1.11	0.44		
15	Calycosin	C16H12O5	20,575-57-9	18	0.52	0.24	-	-
16	Synephrine	C9H13NO2	94-07-5	12	-	-	High	4"Yes"and 1"No"
17	Didymin	C28H34O14	14,259-47-3	11	0.6	0.23	-	-
18	Taxifolin	C15H12O7	480-18-2	10	−0.22	0.27	-	-
19	Loganic acid	C16H24O10	22,255-40-9	8	−0.18	0.4	-	-
20	Rheic acid	C15H8O6	478-43-3	6	−0.19	0.27	-	-
21	β-Lapachone	C15H14O3	4707-32-8	4	-	-	High	5"Yes"
22	2-[(2E)−2-{[5-(4-Bromophenyl)−2-furyl]methylene}hydrazino]−4,6-di(4-morpholinyl)−1, 3,5-triazine	C22H24BrN7O3	9585177	2	-	-	High	4"Yes"and 1"No"
23	Dantron	C14H8O4	117-10-2	2	0.56	0.18	-	-
24	Limonin	C26H30O8	1180-71-8	2	−0.15	0.57	-	-
25	Methylsuccinic acid	C5H8O4	2174-58-5	2	-	-	High	3"Yes"and 2"No"
26	Plumbagin	C11H8O3	481-42-5	2	-	-	High	4"Yes"and 1"No"
27	1, 3,7-Trimethyluric acid	C8H10N4O3	5415-44-1	2	-	-	High	4"Yes"and 1"No"

Moreover, the disease-related targets of CRF were collected from GeneCards, DrugBank, and OMIM, which resulted in 1,854 putative targets. The common targets between the predicted targets of the SSAEP and CRF-related disease were further screened by matching the target information of the SSAEP and CRF-related diseases, resulting in the identification of 139 overlapping targets (Fig. 8B). On this basis, we constructed a protein‒protein interaction (PPI) network and identified core targets by using the CytoHubba plugin. Further visualization and analysis were performed using Cytoscape 3.10.2 platform. In all, 29 crucial molecular targets were obtained after screening (Fig. 8C), and among them were the top 5 hub molecules: TP53, IL6, AKT1, TNF, and ESR1. GO enrichment analysis was carried out to further explore the functional characteristics of the hub genes (Fig. 8D). With respect to biological processes, the targets were mostly involved in cytokine-induced cell responses or cytokine-mediated signaling pathways. At the cellular component level, these genes were related mainly to the transcription factor complex and transcription repressor complex, membrane microdomain, and euchromatin region. The molecular function category was enriched for the biological processes of cytokine activity and cytokine receptor binding. KEGG pathway enrichment also revealed the top 20 related signaling pathways (Fig. 8E), which included several important pathways, cancer pathways, mycobacterial infection mechanisms, AGE-RAGE-mediated pathways of diabetes, hepatitis B viral infection/influenza A viral pathogenesis, and IL-17-mediated signaling cascades.

After the network pharmacology analysis, we selected 5 active ingredients: quercetin, diethyl 4-methoxybenzalmalonate, luteolin, 7-hydroxycoumarin, and 6, 3'-dihydroxy-4, 4'-dimethoxy-5-methylaurone. Molecular docking analysis of these compounds was performed against the five major targets TP53, IL6, AKT1, TNF, and ESR1. The outcome of these interactions is shown in the form of a heatmap (Fig. 8F), with binding energies lower than 0 kJ/mol indicating spontaneous ligand receptor binding, whereas binding energies lower than −5 kJ/mol suggest highly favorable binding interactions. All of the basic compounds in SSAEP were found to have less than −5 kJ/mol for each target, indicating the strong affinity of the active components to their target targets. We further demonstrated the binding interactions of luteolin and quercetin with TNF (Fig. 8G). The binding of luteolin to TNF involves three key amino acid residues (ALA-33, VAL-150, and PHE-144), contributing to a binding energy of −24.85 kJ/mol, whereas quercetin interacts with four key residues (GLY-40, LEU-37, LEU-43, and GLU-42), resulting in a binding energy of −11.84 kJ/mol.

The above results provide further evidence for the joint treatment effect of SSAEP on CRF through the use of network pharmacology and molecular docking methods. The findings demonstrate that SSAEP has a multicomponent effect on different target locations and modulates multiple pathways, providing a theoretical basis for future studies and possible translational applications.

### Quercetin alleviates fibrosis and inflammatory responses in HK-2 cells by inhibiting the IL-17 signaling pathway

**Fig. 9:**
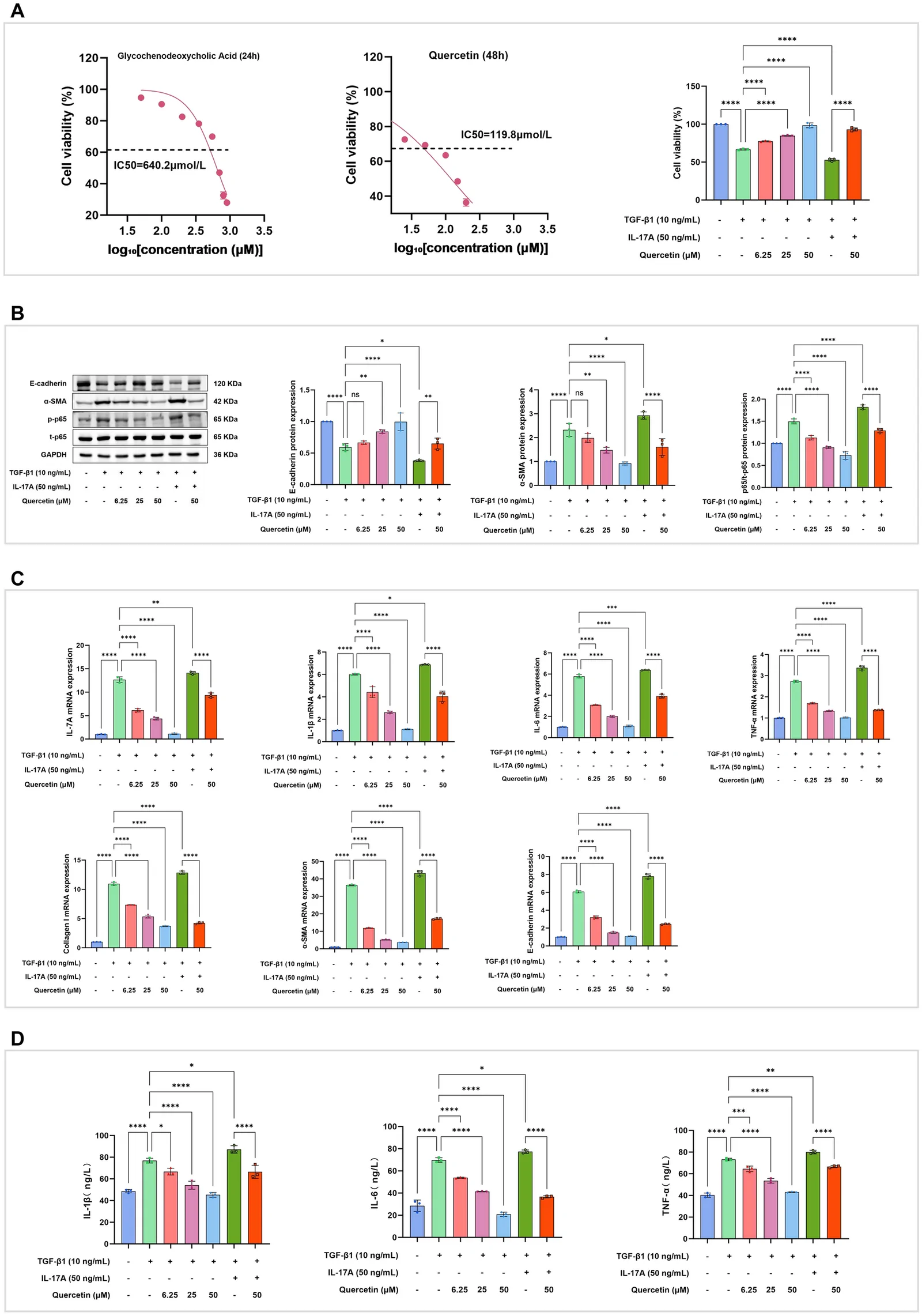
Quercetin alleviated fibrosis and inflammatory responses in HK-2 cells by inhibiting the activity of the IL-17 signaling pathway (^*^P < 0.05, ^**^P < 0.01, ^***^P < 0.001, ^****^P < 0.0001). **A** Assessment of the toxicity of quercetin on HK-2 cells, screening of safe concentrations, and assessment of cell viability via CCK-8 assay. **B** Results of WB assays for each group (E-cadherin, α-SMA, p-p65/t-p65). **C** Results of mRNA expression detection for the qPCR target genes in each group (IL-17RA, IL-1β, IL-6, TNF-α, Collagen I, α-SMA, and E-cadherin). **D** ELISA results for inflammatory factors in the cell supernatant (IL-1β, IL-6, and TNF-α)

To functionally validate these predictions, we conducted a series of in vitro experiments using HK-2 human renal tubular epithelial cells. We first confirmed that quercetin, a major active component of the SSAEP, was nontoxic to HK-2 cells at concentrations up to 50 μM (Fig. 9A). Using a TGF-β1-induced fibrosis model, we found that quercetin treatment dose-dependently rescued cell viability (Fig. 9A). Western blot analysis revealed that quercetin significantly reduced the phosphorylation of NF-κB p65 (p-p65/t-p65 ratio) and reversed the epithelial‒mesenchymal transition (EMT) phenotype by decreasing α-SMA expression and increasing E-cadherin expression (Fig. 9B). At the mRNA level, quercetin downregulated the expression of its receptor, IL-17RA, and suppressed the transcription of proinflammatory cytokines (IL-6, TNF-α, and IL-1β) and fibrotic markers (α-SMA and collagen I) while restoring E-cadherin expression (Fig. 9C). The secretion of inflammatory cytokines (IL-6, TNF-α, and IL-1β) was also significantly inhibited by quercetin (Fig. 9D). Furthermore, the addition of an IL-17 pathway agonist (IL-17A) exacerbated TGF-β1-induced injury, which was effectively reversed by high-dose quercetin (50 μM), confirming that quercetin protects against renal fibrosis and inflammation at least in part by inhibiting the IL-17/NF-κB signaling pathway (Fig. 9D).

### Pathogenicity of the differentially abundant metabolite GCDCA and the antagonistic effect of quercetin

**Fig. 10:**
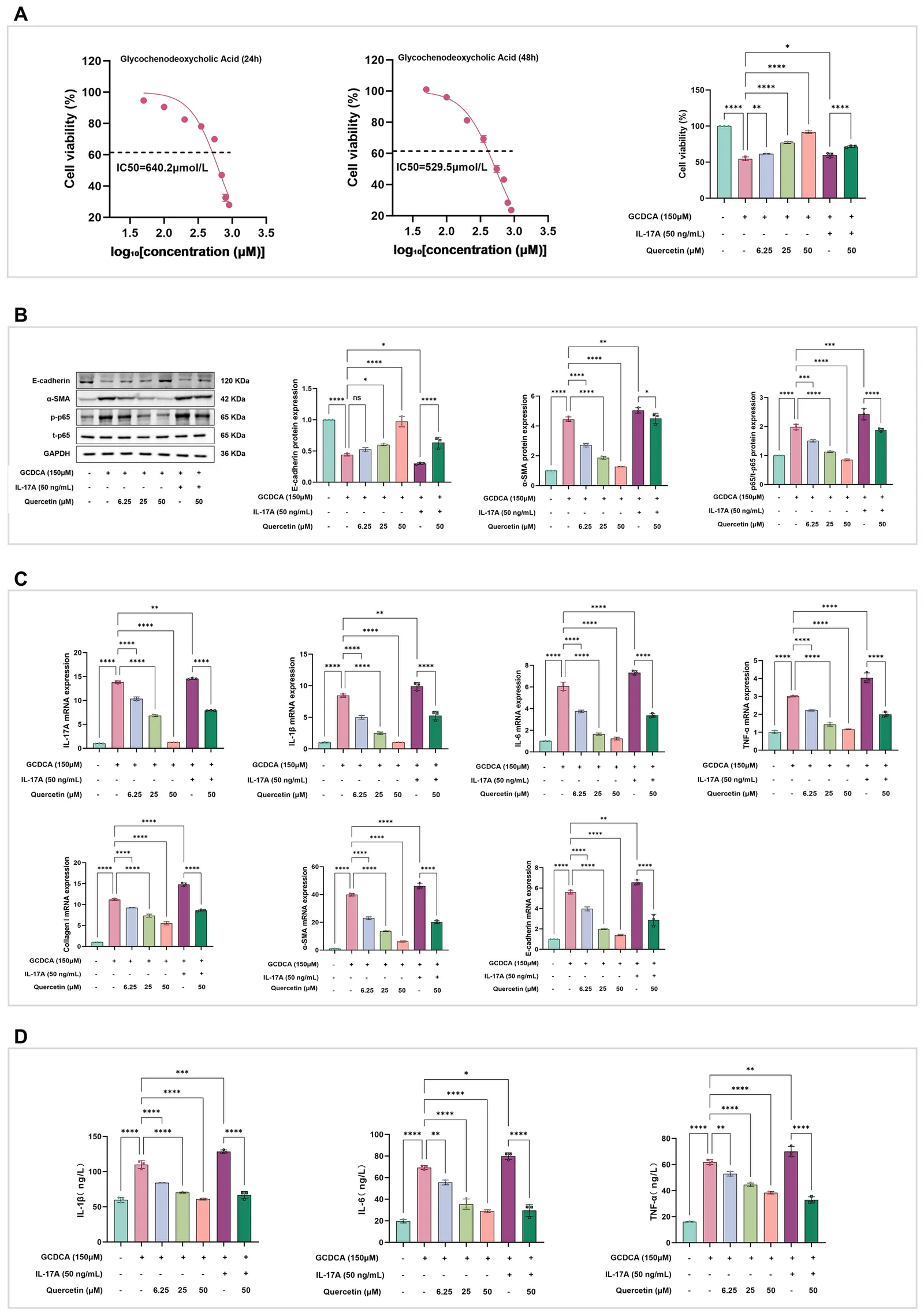
Pathogenicity of the differentially abundant metabolite GCDCA and the antagonistic effect of quercetin (^*^P < 0.05, ^**^P < 0. 01, ^***^P < 0.001, ^****^P < 0.0001). **A** Toxicity of GCDCA to HK-2 cells, screening of safe concentrations, and cell viability assessment using a CCK-8 assay. **B** Results of WB assays for each group (E-cadherin, α-SMA, p-p65/t-p65). **C** Results of mRNA expression detection for the qPCR target genes in each group (IL-17RA, IL-1β, IL-6, TNF-α, Collagen I, α-SMA, and E-cadherin). **D** ELISA results for inflammatory factors in the cell supernatant (IL-1β, IL-6, and TNF-α)

To validate the causal role of the key differentially abundant metabolite GCDCA, we treated HK-2 cells with GCDCA. Following a 24-h exposure, the IC50 value was 640.2 μmol/L; prolonging the exposure to 48 h increased the toxicity, with the IC50 decreasing to 529.5 μmol/L. Treatment with 150 μM GCDCA for 24 h induced consistent and moderate cellular damage (Fig. 10A). GCDCA directly activated the NF-κB pathway, induced a pro-fibrotic EMT phenotype (increased α-SMA expression and decreased E-cadherin expression), and upregulated the expression and secretion of proinflammatory cytokines (Fig. 10B–C). Importantly, cotreatment with the IL-17 agonist further potentiated the cytotoxic and inflammatory effects of GCDCA. Crucially, quercetin effectively attenuated GCDCA-induced injury, restoring cell viability and reversing EMT and inflammatory responses (Fig. 10D). These experiments provide direct evidence that the key metabolite, GCDCA, is a pathogenic driver of CRF progression and that its effects can be mitigated by the active component of SSAEP, thereby supporting the “gut microbiota–metabolite–kidney” axis hypothesis.

### Proposed model of the indirect pharmacological action of the SSAEP against CRF

On the basis of the comprehensive results of 16S rDNA sequencing, metabolomics, and network pharmacology analyses, we propose a comprehensive model to explain the indirect mechanism of action of the SSAEP in improving CRF (*Fig. 11, graphical abstract*). The model reveals a multistep pharmacological cascade:

(1) Direct impacts on the intestinal ecosystem: the bioactive components in the SSAEP (such as quercetin) can directly act on the intestinal environment after being administered via enema.

(2) Intermediate remodeling (gut microbiota): this interaction selectively promotes the growth of certain bacteria (such as *Akkermansia* and *Bacteroidetes*) and inhibits the proliferation of certain bacteria (such as *Clostridium* and *Actinobacteria*), thereby restoring microbial diversity and homeostasis.

(3) Intermediate remodeling stage (gut microbiota-derived metabolites): The reconstructed gut microbiota can regulate the host–microbiota cometabolism process, thereby correcting key metabolite levels (e.g., reducing the levels of glycochenodeoxycholic acid).

(4) Systemic regulation: these modified intermediate metabolites, along with other microbial products, enter the systemic circulation and subsequently modulate host signaling pathways (such as IL-17).

(5) Final treatment outcome: the downstream effects regulated by these pathways include alleviation of systemic and renal inflammatory responses, inhibition of renal fibrosis, and improvement in renal function indicators, thereby collectively delaying the progression of CRF.

This model presents the proposed core concept of indirect pharmacology. That is, the therapeutic endpoint of the SSAEP is achieved through a series of intermediate biological modifications rather than direct effects on renal targets. We emphasize that this model, while strongly supported by the above FMT and omics data, remains a hypothesis requiring direct PK validation.

## Discussion

After SSAEP treatment, the serum levels of BUN and Cys-C, the SCR, and the UA level clearly decreased in the CRF model rats, while pathological changes in kidney tissue were alleviated. These effects were consistent with previous reports that showed that SSAEP could inhibit the development of CRF [18, 20]. Renal fibrosis often accompanies the pathological progression of CRF to ESRD [25, 26], which is characterized by excessive ECM deposition as well as derangement in the renal parenchymal structure [27, 28]. The results of this study confirm that treatment with SSAEP significantly improved kidney disease, as evidenced by decreased collagen deposition as determined by Masson’s staining. The expression levels of alpha smooth muscle actin and Col-1 also decreased, with higher expression of E-cadherin. These results indicate that SSAEP therapy could improve renal function indices and inhibit the occurrence of kidney fibrosis at the level of its pathogenesis mechanism. Notably, in the Ab group and the Ab + SSAEP group, the renoprotective effect of SSAEP was significantly weakened. The results of the FMT experiment revealed that after administration of the SSAEP, kidney function indicators and fibrosis levels significantly improved in the CRF-R group compared with those before the administration. Taken together, these results suggest that the effect of the SSAEP is at least partly dependent on an intact microbiota, whose mode of action is strongly dependent on the “gut–kidney axis”. This dependency provides strong correlational and causal (via FMT) support for the proposed microbiome-dependent mechanism, in which the microbiota serves as a key intermediate mediator.

Recently, a new theory called “the gut–kidney axis” has been proposed to explain the pathogenesis of CRF. The disruption of mucosal tight junction proteins and dysbiosis in the gut caused by CRF allows for entry into the blood stream of uremic solutes such as indole-3-sulfonate and para-cresol sulfonate as well as lipopolysaccharides from gram-negative bacteria and later induces chronic low-grade inflammation and oxidative damage, exacerbating renal tissue damage and fibrosis development [29]. The results of our microbiome analysis provide experimental evidence to substantiate the above hypothesis: we observed a significant decrease in the alpha and beta diversity of the intestinal flora from rats with CRF, which is consistent with the results of several clinical studies as well as preclinical models that have reported reduced diversity associated with CRF [30–32]. SSAEP treatment resulted in significant improvement in the microbial diversity, and the results of the bacterial population analysis revealed that SSAEP treatment significantly increased the abundance of *Bacteriodota* and *Akkermansia* whereas it decreased the abundance of *Actinomycetota*, *Clostridium*, and *Corynebacterium* species. *Akkermansia*, the other broadly reported beneficial bacteria that produce metabolites, including SCFAs (e.g., butyrate), demonstrate anti-inflammatory characteristics and help maintain gut barrier function [33]. The increase in the relative abundance of *Akkermansia* was negatively correlated with kidney injury markers, suggesting that this taxon may be an important mediator of the therapeutic benefits of SSAEP. Conversely, a higher relative abundance of *Clostridium* has also been associated with gut inflammation and the production of potentially detrimental metabolites in other disease contexts [34]. SSAEP affects the intestinal microflora by decreasing the relative abundance of certain taxa with reported pathogenic potential (e.g., *Clostridium* and *Corynebacterium*) and increasing the relative abundance of taxa commonly associated with gut health (e.g., *Akkermansia* and *Allobaculum*). This modulation of specific microbial groups contributes to the restoration of gut ecological balance and the improvement of gut–kidney axis communication. This ability to alter bacterial flora seems critical for normalizing the function of this important physiological interface.

Metabolomic analysis links microbiota changes to the host phenotype and identifies key intermediate substances. In this study, we identified three key SSAEP-regulated metabolites: pyridoxic acid, 3-hydroxyanthranilic acid, and glycochenodeoxycholic acid. Notably, 4-pyridoxic acid was identified as a terminal metabolite of vitamin B6. Our study and previous studies [35] report higher levels in patients with CRF and inflammation, with strong positive correlations with renal disease indicators and proinflammatory cytokines. Nevertheless, the exact biological role(s) of this compound remain a topic of debate. 4-Pyridoxic acid might act as a secondary oxidant product of CRF, but it may also have other signaling activities [36]. 3-Hydroxyanthranilic acid is the intermediary metabolic product of tryptophan degradation and is associated with inflammation and immune regulation [37]. Glycochenodeoxychic acid is a secondary bile acid derived from primary bile acids by conversion by intestinal bacteria [38]. Bile acid metabolism is a paradigmatic example of an intimate relationship between the host and microbiota, with its imbalance being implicated in multiple chronic inflammatory conditions [39]. In our study, we observed a significant increase in the levels of all three metabolites in the CRF group, which returned to normal levels after SSAEP therapy. In particular, these changes were positively correlated with the relative abundance of taxa associated with proinflammatory states in the context of CRF (e.g., *Clostridium*, *Corynebacterium*, and *Actinomycetota*), and they were negatively correlated with the relative abundance of taxa often linked to gut barrier integrity and anti-inflammatory effects (e.g., *Akkermansia* and *Bacteroidota*). These correlation patterns support the hypothesis that SSAEP may ameliorate uremic conditions by modulating these microbial–metabolite axes. Crucially, these metabolites act as intermediate substances, translating information from gut microbiota dysbiosis into systemic signals that drive renal pathology. The regulation of its level by SSAEP is a key step in its indirect action cascade reaction.

The network pharmacology findings predicted that SSAEP acts through multiple pathways, with quercetin as a key component. Our subsequent in vitro experiments provide direct functional evidence to support these predictions. The results demonstrate that quercetin, a major bioactive compound in SSAEP, functionally validates the predicted role of the IL-17/NF-κB signaling pathway. Quercetin dose-dependently inhibited TGF-β1-induced EMT and inflammatory responses in HK-2 cells, but these effects were negated by an IL-17 pathway agonist. These findings confirm the role of the IL-17 pathway, identified through our KEGG analysis, as a core downstream mechanism for the anti-fibrotic and anti-inflammatory effects of SSAEP [40]. In addition, the ongoing interaction between AGEs and their cell surface receptor RAGE sustains proinflammatory/fibrotic signals in CRF [41–43]. These findings align with the known antifibrotic properties of flavonoids [44, 45] and provide an experimental link between the predicted targets and observed renal protection. Furthermore, the findings reveal a critical missing link in the proposed “gut microbiota–metabolite–kidney” axis. By exogenously administering the key differentially abundant metabolite GCDCA to HK-2 cells, we demonstrated its direct pathogenic role. GCDCA directly induced cell injury, inflammation, and fibrosis in vitro, resulting in the replication of key features of CRF. These observations are consistent with the reported role of deregulated bile acid metabolism under inflammatory conditions [38, 39]. Importantly, quercetin effectively antagonized GCDCA-induced injury, demonstrating that the active components of SSAEP can counteract the harmful effects of the very metabolites whose levels are reduced in vivo. This reciprocal validation strongly supports the causal chain from gut microbiota dysbiosis → altered metabolite production (e.g., GCDCA) → direct renal tubular injury and explains how SSAEP, by reshaping the microbiota, breaks this cycle. Collectively, the findings of the in vitro experiments provide robust causal evidence for the proposed indirect pharmacological model. The validation of both the active ingredient (quercetin) and a key pathogenic metabolite (GCDCA) and their interaction with the IL-17 pathway substantially strengthens our mechanistic conclusions.

This study has several limitations. Most importantly, while the findings strongly support a mechanism that is dependent on the gut microbiota, direct PK evidence that could entirely rule out the possibility that some SSAEP components may be absorbed and exert direct effects on the kidney is lacking. The proposed “indirect pharmacology” hypothesis posits that the active components of SSAEP, such as luteolin and quercetin, act primarily on the intestinal epithelium or the gut microbiota. To directly test this hypothesis, future studies should quantify the concentrations of the major active compounds of SSAEP in the plasma and kidney tissue of treated rats. Such experiments are critical to definitively establish whether the mechanism is exclusively indirect. Without these PK data, our model, although compelling and strongly supported by FMT data, remains a well-supported working hypothesis that requires further validation.

## Conclusion

In summary, this study elucidates the mechanism through which SSAEP treats CRF from an indirect pharmacological perspective. The findings provide strong evidence indicating that the mechanism of action of SSAEP is likely not achieved through direct targeting of the kidneys alone but rather through a multistep mode of action that is heavily dependent on the gut microbiota and its metabolites. This study revealed that SSAEP first remodels the gut microbiota ecosystem, increasing the abundance of certain bacteria (such as *Akkermansia*) and inhibiting the abundance of certain bacteria (such as *Clostridium*). This microbial reconstruction leads to the normalization of levels of key microbially derived intermediate metabolites (such as GCDCA) — substances that are abnormally elevated in CRF and associated with disease severity. These systemic metabolic changes subsequently lead to the downregulation of key proinflammatory and profibrotic pathways (such as the IL-17 signaling pathway). Ultimately, SSAEP improved renal function, reduced inflammation, and inhibited the fibrosis process in CRF rats.

The model proposed in this study (*Fig. 11*, *graphical abstract*) integrates the above findings into a coherent proposed microbiome-dependent framework: SSAEP components → gut microbiota → microbiota-derived metabolites → systemic signaling pathways → CRF improvement. We emphasize that this model, while robustly supported by our FMT and omics data, remains a hypothesis that requires direct PK validation to confirm whether the effects are exclusively indirect. Our in vitro experiments provide direct functional evidence for this cascade, confirming that the active component quercetin acts by inhibiting the IL-17/NF-κB pathway and that the key metabolite, GCDCA, acts as a direct pathogenic driver. This reciprocal validation strongly supports the causal chain of the “gut microbiota–metabolite–kidney” axis. This study not only provides a modern interpretation of the traditional Chinese medicine theory of “regulation of bowel movement and removal of turbid qi” by defining relevant biological associations within the gut–kidney axis but also offers a strategic blueprint for developing novel therapies for CRF.

## Supplementary Information

Supplementary Material 1

Supplementary Material 2

Supplementary Material 3

## Data Availability

No datasets were generated or analysed during the current study.

## Abbreviations

Ab: Antibiotic
Ab + SSAEP: Antibiotic combined with SSAEP
AGE-RAGE: Receptor for advanced glycation end products
AKT1: AKT serine/threonine kinase 1
BP: Biological Processes
BSA: Bovine serum albumin
CC: Cellular Components
CCK-8: Cell Counting Kit-8
CKD: Chronic kidney disease
CNKI: China National Knowledge Infrastructure
CRF: Chronic renal failure
CRF-R: The fecal transplantation CRF group
Ctrl: Control
Cys-C: Cystatin c
DDA: Data-dependent acquisition
DL: Drug-likeness
ECM: Extracellular matrix
ELISA: Enzyme-linked immunosorbent assay
ESI: Electrospray ionization
ESRD: End-stage renal disease
FBS: Fetal bovine serum
FMT: Fecal microbiota transplantation
GCA: Glycocholic acid
GCDCA: Glycochenodeoxycholic acid
GO: Gene Ontology
HE: Hematoxylin and eosin
HK-2: Human renal proximal tubular epithelial cells
HRP: Horseradish peroxidase
IL-17: Interleukin-17
IL‑17RA: IL‑17 receptor A
IL-1β: Interleukin-1β
IL-6: Interleukin-6
IL-6: Interleukin-6
KEGG: Kyoto Encyclopedia of Genes and Genomes
KWI: Kidney weight index
LC‒MS/MS: Liquid chromatography-tandem mass spectrometry
LDA: Linear Discriminant Analysis
MF: Molecular Functions
IHC: Immunohistochemistry
OPLS-DA: Orthogonal partial least squares-discriminant analysis
OUT: Operational taxonomic unit
PCoA: Principal co-ordinates analysis
PPI: Protein‒protein interaction
PK: Pharmacokinetic
SCR: Creatinine
qPCR: Quantitative real-time PCR
SD: Sprague Dawley
SPF: Security processing facility
SRA: SEQUENCE Read Archive
SSAEP: Shenshuai 'an enema prescription
SSAEP_L: SSAEP low-dose
SSAEP_H: SSAEP high-dose
SSAEP-R: The fecal transplantation SSAEP group
SUN: Serum urea nitrogen
TCM: Traditional Chinese medicine
TIC: Total ion chromatograms
TNF: Tumor necrosis factor
TNF-α: Tumor necrosis factor-α
TNF-β: Tumor necrosis factor-β
TP53: Tumor protein p53
UA: Uric acid
WB: Western blotting
Wt: Weight
