# Supplementary material for: Network pharmacology, gut microbiota, and metabolomics integration reveal that Shenshuai 'an enema prescription (SSAEP) improves CRF mechanisms by reshaping the gut microbiota and correcting host–microbe cometabolism
Source: Chin Med. 2026 Nov 13;21:239. doi: 10.1186/s13020-026-01484-z (PMC13760777; doi:10.1186/s13020-026-01484-z)
Supplement: Supplementary file 1 [file 13020_2026_1484_MOESM1_ESM.docx]

**Table S1.** Chemical composition of the 82 principal compounds identified in the SSAEP.

| **No.** | **RT**  **(min)** | **Peak Intensity** | **Annot. DeltaMass [ppm]^*^** | **Calc. MW^#^** | **Molecular formula** | **Identity** | **CAS** | **Class** | **Relative concentration (μg/mL)^△^** |
| --- | --- | --- | --- | --- | --- | --- | --- | --- | --- |
| 1 | 1.146 | 16998932146 | -4.58 | 146.02085 | C5H6O5 | alpha-Ketoglutaric acid | 328-50-7 | Keto acids and derivatives | 13645.64589 |
| 2 | 5.322 | 11256385914 | -0.3 | 354.09497 | C16H18O9 | 1-Caffeoylquinic acid | 928005-87-2 | Organooxygen compounds | 9035.900307 |
| 3 | 4.442 | 10155391715 | -0.851 | 354.0949 | C16H18O9 | Cryptochlorogenic acid | 905-99-7 | Organooxygen compounds | 8152.093205 |
| 4 | 6.814 | 9756249468 | -0.7 | 450.1159 | C21H22O11 | Astilbin | 29838-67-3 | Flavonoids | 7831.687563 |
| 5 | 12.818 | 8527638904 | -0.88 | 266.13045 | C18H18O2 | Magnolol | 528-43-8 | Benzene and substituted derivatives | 6845.438277 |
| 6 | 0.828 | 4954983040 | -9.72 | 210.07325 | C8H10N4O3 | 1, 3,7-Trimethyluric acid | 5415-44-1 | Imidazopyrimidines | 3977.540671 |
| 7 | 2.875 | 4722770848 | -7.01 | 132.04133 | C5H8O4 | Methylsuccinic acid | 2174-58-5 | Fatty Acyls | 3791.135706 |
| 8 | 7.717 | 4514360870 | -0.2 | 434.12121 | C21H22O10 | Engeletin | 572-31-6 | Flavonoids | 3623.837623 |
| 9 | 7.97 | 3751159560 | -0.32 | 452.11059 | C24H20O9 | 9, 10-Dihydro-10- (3,4-dihydroxyphenyl) -pyrano [ 2,3-h ] catechin-8-one | 85081-24-9 | Flavonoids | 3011.188855 |
| 10 | 9.835 | 3675806447 | -0.51 | 272.06834 | C15H12O5 | Naringenin | 480-41-1 | Flavonoids | 2950.700239 |
| 11 | 10.946 | 3359534094 | -0.65 | 470.19376 | C26H30O8 | Limonin | 1180-71-8 | Prenol lipids | 2696.817201 |
| 12 | 6.914 | 3134526530 | -1.44 | 900.23286 | C44H52O12S4 | 2, 2′-{[26, 28-Dihydroxy-5, 11, 17, 23-tetrakis(2-methyl-2-propanyl)-2, 8, 14, 20-tetraoxido-2, 8, 14, 20-tetrathiapentacyclo[19. 3. 1. 1~3, 7~. 1~9, 13~. 1~15, 19~]octacosa-1(25), 3(28), 4, 6, 9(27), 10, 12, 15(26), 16, 18, 21, 2 3-dodecaene-25, 27-diyl]bis(oxy)}diacetic acid | NA | Benzene and substituted derivatives | 2516.195646 |
| 13 | 6.133 | 2971469242 | -0.37 | 404.13171 | C17H24O11 | Secoxyloganin | 58822-47-2 | Prenol lipids | 2385.303777 |
| 14 | 12.273 | 2815751623 | -0.65 | 266.13051 | C18H18O2 | Honokiol | 35354-74-6 | Benzene and substituted derivatives | 2260.30372 |
| 15 | 7.076 | 2794332785 | -0.17 | 580.1791 | C27H32O14 | Naringin | 10236-47-2 | Flavonoids | 2243.110059 |
| 16 | 1.831 | 2545076178 | -4.41 | 170.02077 | C7H6O5 | Gallic acid | 149-91-7 | Benzene and substituted derivatives | 2043.022938 |
| 17 | 11.074 | 2443985298 | -0.63 | 284.03191 | C15H8O6 | Rheic acid | 478-43-3 | Anthracenes | 1961.873702 |
| 18 | 4.814 | 2197292023 | -0.63 | 376.13671 | C16H24O10 | Loganic acid | 22255-40-9 | Prenol lipids | 1763.844258 |
| 19 | 9.95 | 2153831073 | -0.57 | 330.24044 | C18H34O5 | (−)-pinellic acid | 97134-11-7 | Fatty Acyls | 1728.956612 |
| 20 | 2.176 | 2070090883 | -3.92 | 192.06264 | C7H12O6 | Quinic acid | 77-95-2 | Organooxygen compounds | 1661.735391 |
| 21 | 1.337 | 2057487434 | -5.6 | 148.03634 | C5H8O5 | δ-Ribono-1,4-lactone | 3327-63-7 | Organooxygen compounds | 1651.618155 |
| 22 | 8.755 | 1920451187 | 0.15 | 594.19494 | C28H34O14 | Poncirin | 14941-08-3 | Flavonoids | 1541.614298 |
| 23 | 12.304 | 1694755823 | -1.41 | 270.05244 | C15H10O5 | Emodin | 518-82-1 | Anthracenes | 1360.440623 |
| 24 | 3.856 | 1511069223 | -5.4 | 154.02578 | C7H6O4 | Protocatechuic acid | 99-50-3 | Benzene and substituted derivatives | 1212.988872 |
| 25 | 7.129 | 1474748260 | -2.06 | 513.11134 | C22H24BrN7O3 | 2-[(2E)-2-{[5-(4-Bromophenyl)-2-furyl]methylene}hydrazino]-4,6-di(4-morpholinyl)-1, 3,5-triazine | NA | Triazines | 1183.832747 |
| 26 | 1.434 | 1457554562 | -4.34 | 160.03648 | C6H8O5 | alpha-ketoadipic acid | 3184-35-8 | Keto acids and derivatives | 1170.030755 |
| 27 | 10.525 | 1445737782 | -0.79 | 268.07335 | C16H12O4 | Formononetin | 485-72-3 | Isoflavonoids | 1160.545007 |
| 28 | 1.148 | 1135464427 | -9.1 | 112.01502 | C5H4O3 | 3-Furoic acid | 488-93-7 | Furans | 911.4775777 |
| 29 | 8.988 | 978967224.9 | -0.69 | 432.10535 | C21H20O10 | Emodin-8-O-β-D-glucopyranoside | 23313-21-5 | Anthracenes | 785.8517215 |
| 30 | 5.362 | 738967311.9 | -0.95 | 374.12094 | C16H22O10 | Geniposidic acid | 27741-01-1 | Prenol lipids | 593.1952771 |
| 31 | 0.956 | 732577815.7 | -5.87 | 136.03637 | C4H8O5 | L-THREONIC ACID | 7306-96-9 | Organooxygen compounds | 588.0662018 |
| 32 | 8.855 | 705424760.7 | -0.49 | 408.14183 | C20H24O9 | Tinnevellin glucoside | 80358-06-1 | Organooxygen compounds | 566.2694812 |
| 33 | 3.862 | 625106907 | -9.22 | 110.03576 | C6H6O2 | Catechol | 120-80-9 | Phenols | 501.7954907 |
| 34 | 5.793 | 575707640.6 | -4.17 | 180.04151 | C9H8O4 | Caffeic acid | 331-39-5 | Cinnamic acids and derivatives | 462.140947 |
| 35 | 5.612 | 557146758.5 | -3.32 | 192.02637 | C6H8O7 | Citric acid | 77-92-9 | Carboxylic acids and derivatives | 447.2414685 |
| 36 | 6.642 | 388356573.8 | 0.13 | 596.1742 | C27H32O15 | Eriocitrin | 13463-28-0 | Flavonoids | 311.7475992 |
| 37 | 4.442 | 380763640 | -0.59 | 462.17345 | C20H30O12 | Forsythoside E | 93675-88-8 | Organooxygen compounds | 305.6524818 |
| 38 | 1.828 | 347562474.8 | -7.29 | 126.03078 | C6H6O3 | Pyrogallol | 87-66-1 | Phenols | 279.0007288 |
| 39 | 11.214 | 34319993208 | -2.53 | 402.13045 | C21H22O8 | Nobiletin | 478-01-3 | Flavonoids | 259.7709784 |
| 40 | 11.068 | 310323108.2 | -1.85 | 240.04181 | C14H8O4 | Dantron | 117-10-2 | Anthracenes | 249.1073681 |
| 41 | 11.726 | 32711900690 | -2.8 | 372.11986 | C20H20O7 | Tangeretin | 481-53-8 | Flavonoids | 247.5991879 |
| 42 | 9.102 | 294931907.9 | -0.93 | 302.04237 | C15H10O7 | Quercetin | 117-39-5 | Flavonoids | 236.7523055 |
| 43 | 2.317 | 290993182 | -4.63 | 165.07821 | C9H11NO2 | L-Phenylalanine | 150-30-1 | Carboxylic acids and derivatives | 233.5905505 |
| 44 | 8.002 | 27599623389 | -2.18 | 278.11482 | C15H18O5 | Diethyl 4-methoxybenzalmalonate | 6768-23-6 | Cinnamic acids and derivatives | 208.9039216 |
| 45 | 10.908 | 21292806173 | -2.55 | 260.1042 | C15H16O4 | Isomeranzin | 1088-17-1 | Coumarins and derivatives | 161.16708 |
| 46 | 0.902 | 20002531858 | 1.74 | 103.09989 | C5H13NO | Choline | 62-49-7 | Organonitrogen compounds | 151.4008828 |
| 47 | 1.121 | 16316888037 | -1.59 | 149.08383 | C9H11NO | N-(2,4-Dimethylphenyl)formamide | 60397-77-5 | Benzene and substituted derivatives | 123.503928 |
| 48 | 1.278 | 15740799290 | -2.27 | 267.09615 | C10H13N5O4 | Adenosine | 58-61-7 | Purine nucleosides | 119.1434627 |
| 49 | 2.273 | 14602397137 | 0.27 | 102.04698 | C8H6 | Phenylacetylene | 536-74-3 | Benzene and substituted derivatives | 110.526799 |
| 50 | 4.601 | 125675859.5 | -2.82 | 204.0893 | C11H12N2O2 | L-Tryptophan | 73-22-3 | Indoles and derivatives | 100.8844709 |
| 51 | 4.559 | 13237368771 | 3.21 | 490.17894 | C24H30N2O7S | Ethyl 1-[N-(3,4-dimethoxyphenyl)-N-(phenylsulfonyl)glycyl]-4-piperidinecarboxylate | NA | Benzene and substituted derivatives | 100.194782 |
| 52 | 9.644 | 117851558.5 | -0.58 | 446.12104 | C22H22O10 | Glycitin | 40246-10-4 | Isoflavonoids | 94.60362693 |
| 53 | 6.676 | 98559586.96 | -4.64 | 164.04658 | C9H8O3 | p-Coumaric acid | 501-98-4 | Cinnamic acids and derivatives | 79.11727697 |
| 54 | 1.242 | 95746825.46 | -0.51 | 363.05781 | C10H14N5O8P | Guanosine monophosphate (GMP) | 85-32-5 | Purine nucleotides | 76.85937353 |
| 55 | 9.039 | 9746031479 | -2.7 | 284.06771 | C16H12O5 | Calycosin | 20575-57-9 | Isoflavonoids | 73.76854994 |
| 56 | 6.095 | 85757819.98 | 0 | 610.18977 | C28H34O15 | Hesperidin | 520-26-3 | Flavonoids | 68.84084446 |
| 57 | 5.268 | 8733128625 | -2.33 | 354.09426 | C16H18O9 | Chlorogenic acid | 327-97-9 | Organooxygen compounds | 66.10180117 |
| 58 | 6.887 | 8051068312 | -2.72 | 304.05747 | C15H12O7 | Taxifolin | 480-18-2 | Flavonoids | 60.93922804 |
| 59 | 1.518 | 7798760598 | -1.79 | 151.09944 | C9H13NO | Cathine | 492-39-7 | Benzene and substituted derivatives | 59.02948927 |
| 60 | 0 74 | 57407704.92 | -4.95 | 155.06871 | C6H9N3O2 | L-Histidine | 4998-57-6 | Carboxylic acids and derivatives | 46.08320135 |
| 61 | 1.257 | 53804441.93 | -3.77 | 181.07321 | C9H11NO3 | L-Tyrosine | 556-03-6 | Carboxylic acids and derivatives | 43.19073432 |
| 62 | 5.266 | 5579282652 | -1.69 | 162.03142 | C9H6O3 | 7-Hydroxycoumarine | 93-35-6 | Coumarins and derivatives | 42.23006993 |
| 63 | 6.13 | 5494469109 | -1.57 | 182.05762 | C9H10O4 | Veratric acid | 93-07-2 | Benzene and substituted derivatives | 41.58810894 |
| 64 | 6.129 | 4771235729 | -1.72 | 150.03144 | C8H6O3 | Phenylglyoxylic acid | 611-73-4 | Benzene and substituted derivatives | 36.11389332 |
| 65 | 10.708 | 4751415108 | -2.64 | 372.11992 | C20H20O7 | Sinensetin | 2306-27-6 | Flavonoids | 35.96386933 |
| 66 | 7.87 | 4679579716 | -2.18 | 430.12544 | C22H22O9 | Ononin | 486-62-4 | Isoflavonoids | 35.42014108 |
| 67 | 12.332 | 4649142139 | -2.53 | 328.09386 | C18H16O6 | 6, 3'-Dihydroxy-4, 4'-dimethoxy-5-methylaurone | NA | Aurone flavonoids | 35.18975645 |
| 68 | 11.796 | 4266662743 | -2.82 | 418.1252 | C21H22O9 | 3'-C-Glucosylisoliquiritigenin | NA | Linear 1,3-diarylpropanoids | 32.29473702 |
| 69 | 0.987 | 3718992879 | -1.84 | 137.04743 | C7H7NO2 | Trigonelline HCl | 6138-41-6 | Pyridines and derivatives | 28.14937675 |
| 70 | 1.087 | 3086429137 | -2.45 | 113.05863 | C4H7N3O | Creatinine | 60-27-5 | Carboxylic acids and derivatives | 23.36144742 |
| 71 | 8.001 | 2975026076 | -2.11 | 242.09378 | C15H14O3 | β-Lapachone | 4707-32-8 | Naphthopyrans | 22.51822807 |
| 72 | 7.095 | 2776359043 | -1.87 | 580.17812 | C27H32O14 | Narirutin | 14259-46-2 | Flavonoids | 21.01450023 |
| 73 | 6.129 | 2207522483 | -2.23 | 224.06797 | C11H12O5 | Sinapic acid | 530-59-6 | Cinnamic acids and derivatives | 16.70892741 |
| 74 | 8 | 2140942376 | -1.91 | 188.04698 | C11H8O3 | Plumbagin | 481-42-5 | Naphthalenes | 16.20497686 |
| 75 | 1.123 | 2127826740 | -1.83 | 167.09432 | C9H13NO2 | Synephrine | 94-07-5 | Phenols | 16.10570348 |
| 76 | 8.763 | 2036204768 | -2.03 | 594.19365 | C28H34O14 | Didymin | 14259-47-3 | Flavonoids | 15.4122089 |
| 77 | 10.191 | 1930409847 | -2.88 | 372.11983 | C20H20O7 | Isosinensetin | 17290-70-9 | Flavonoids | 14.61143805 |
| 78 | 12.372 | 1586408770 | -2.48 | 388.11485 | C20H20O8 | Artemetin | 479-90-3 | Flavonoids | 12.00766433 |
| 79 | 9.021 | 1246275535 | -3.55 | 286.04672 | C15H10O6 | Luteolin | 491-70-3 | Flavonoids | 9.433166643 |
| 80 | 1.118 | 826308345.5 | -0.63 | 123.03195 | C6H5NO2 | Nicotinic acid | 59-67-6 | Pyridines and derivatives | 6.254398889 |
| 81 | 1.34 | 249244893.8 | -1.21 | 136.03835 | C5H4N4O | Hypoxanthine | 68-94-0 | Imidazopyrimidines | 1.886556023 |
| 82 | 6.919 | 174924189.9 | -1.6 | 594.15752 | C27H30O15 | Kaempferol-3-O-rutinoside | 17650-84-9 | Flavonoids | 1.324016228 |

Note: *Detection bias of the reference compound; ^#^The detected molecular weight value; ^△^Relative concentration of each substance in the SSAEP sample.
