# Supplementary material for: Network pharmacology, gut microbiota, and metabolomics integration reveal that Shenshuai 'an enema prescription (SSAEP) improves CRF mechanisms by reshaping the gut microbiota and correcting host–microbe cometabolism
Source: Chin Med. 2026 Nov 13;21:239. doi: 10.1186/s13020-026-01484-z (PMC13760777; doi:10.1186/s13020-026-01484-z)
Supplement: Supplementary file 2 [file 13020_2026_1484_MOESM2_ESM.docx]

**Table S2.** A total of 113 differentially abundant metabolites were identified in both serum samples and colonic content samples across all groups.

| **No.** | **Metab ID** | **Metabolite(s)** | **Metabolite type** | **Serum samples** | | | **Colonic contents samples** | | | **Microbial community-associated metabolites**  **(yes or no)** |
| --- | --- | --- | --- | --- | --- | --- | --- | --- | --- | --- |
|  |  |  |  | **VIP** | **CRF_vs_Ctrl** | **SSAEP_H_vs_CRF** | **VIP** | **CRF_vs_Ctrl** | **SSAEP_H_vs_CRF** |  |
| 1 | metab_13493 | Val-Leu-Ser | Tripeptide | 3.408 | up | down | 2.2355 | down | up | yes |
| 2 | metab_10796 | Hexadeca-7, 10, 13-trienoic acid | Polyunsaturated fatty acid | 3.1073 | up | down | 1.3049 | down | up | no |
| 3 | metab_11642 | Strophanthidin | Cardiac glycoside aglycone | 3.0778 | up | down | 2.0858 | up | down | no |
| 4 | metab_13333 | Ile-Hyp | Dipeptide | 3.0107 | up | down | 1.0641 | up | down | yes |
| 5 | metab_2721 | Lys-Tyr | Dipeptide | 2.7274 | up | down | 2.0188 | down | up | yes |
| 6 | metab_2514 | Ser-Leu-Pro | Tripeptide | 2.6802 | up | down | 1.5323 | down | up | yes |
| 7 | metab_17222 | 2-Coumaroylquinic acid | Phenylpropanoid conjugate | 2.5959 | up | down | 2.0576 | down | up | no |
| 8 | metab_13292 | Trp-Lys | Dipeptide | 2.4175 | up | down | 1.233 | down | up | yes |
| 9 | metab_17144 | Bergenin | Phenylpropanoid glycoside | 2.3597 | up | down | 2.1729 | down | up | no |
| 10 | metab_1756 | 5-methoxy methylone | Cathinone derivative | 2.3491 | up | down | 1.8683 | up | down | no |
| 11 | metab_3046 | Desoxycortisone acetate | Corticosteroid ester | 2.3469 | up | down | 2.174 | up | down | no |
| 12 | metab_24860 | 3-Hydroxyindolin-2-one-sulfate | Sulfated oxindole derivative | 2.3389 | up | down | 2.2217 | down | up | yes |
| 13 | metab_18413 | 6,8-dihydroxy-7-methoxy-3-methyl-3,4-dihydroisochromen-1-one | Dihydroxy methoxy isochromenone | 2.3347 | up | down | 1.8765 | down | up | no |
| 14 | metab_18794 | 4, 4'-Dihydroxytruxillic acid | Dihydroxy aromatic carboxylic acid | 2.3266 | up | down | 1.5665 | down | up | no |
| 15 | metab_19502 | Ulimorelin | Ghrelin receptor agonist | 2.3227 | up | down | 1.5895 | up | down | no |
| 16 | metab_13942 | 3-Hydroxyanthranilic acid | Hydroxy anthranilic acid derivative | 2.3214 | up | down | 1.9578 | up | down | yes |
| 17 | metab_2672 | 4-[(2,4-Dihydroxy-3,3-dimethylbutanoyl)amino]butanoic acid | Hydroxybutanoyl amino butanoic acid | 2.2461 | up | down | 2.1809 | down | up | no |
| 18 | metab_22144 | N-Arachidonoyl Proline | Fatty acid amino acid conjugate | 2.235 | up | down | 1.748 | up | down | no |
| 19 | metab_18117 | Coumafuryl | Coumarin derivative | 2.213 | up | down | 1.6141 | down | up | no |
| 20 | metab_23234 | Asp-Leu-Ser-Glu | Tetrapeptide | 2.1698 | up | down | 2.1397 | up | down | yes |
| 21 | metab_18275 | Hematommic acid | Lichen carboxylic acid | 2.1494 | up | down | 1.5237 | down | up | no |
| 22 | metab_2667 | Ser-Tyr | Dipeptide | 2.1445 | up | down | 1.6979 | down | up | yes |
| 23 | metab_22591 | Quinolactacin B | Quinolone derivative | 2.1437 | down | up | 1.6648 | down | up | no |
| 24 | metab_11166 | 4-(2-Aminophenyl)-2,4-dioxobutanoic acid | Aminophenyl dioxobutanoic acid | 2.0983 | up | down | 1.5699 | down | up | no |
| 25 | metab_17241 | 5-Hydroxy-6-methoxyindole glucuronide | Hydroxy methoxy indole glucuronide | 2.0776 | up | down | 1.5532 | down | up | yes |
| 26 | metab_10764 | 15-keto Latanoprost (free acid) | Prostanoid | 2.0754 | up | down | 2.7107 | up | down | no |
| 27 | metab_22495 | Diethyl phthalic acid | Phthalate ester | 2.0733 | up | down | 2.067 | up | down | no |
| 28 | metab_18133 | Indicaxanthin | Betalain | 2.025 | up | down | 2.9758 | down | up | no |
| 29 | metab_13106 | 2-[(2-hydroxy-3-methylbutanoyl)amino]-3-methylbutanoic acid | Hydroxybutanoyl amino methylbutanoic acid | 2.0201 | up | down | 1.444 | up | down | no |
| 30 | metab_24626 | 9-[3,4-dihydroxy-5-(methoxymethyl)oxolan-2-yl]-1H-purin-6-one | Purine oxolane derivative | 2.0143 | up | down | 1.4409 | down | up | no |
| 31 | metab_1477 | 4-pyridoxic acid | Pyridoxine metabolite | 1.9437 | up | down | 1.1769 | up | down | yes |
| 32 | metab_23074 | 2-(2-Hydroxyethyl)phenyl hydrogen sulfate | Sulfated hydroxyethylphenyl derivative | 1.9393 | up | down | 2.298 | up | down | no |
| 33 | metab_2054 | 2,5-dihydroxybenzaldehyde | Dihydroxy aromatic aldehyde | 1.9352 | up | down | 1.9702 | down | up | no |
| 34 | metab_15511 | Allysine | Lysine derivative | 1.9047 | up | down | 1.3597 | down | up | no |
| 35 | metab_17239 | 2-[3-(sulfooxy)phenyl]acetic acid | Sulfated phenylacetic acid | 1.8977 | up | down | 1.6078 | up | down | no |
| 36 | metab_1976 | Val-Gly-Tyr | Tripeptide | 1.8891 | up | down | 1.6891 | down | up | yes |
| 37 | metab_19331 | Elymoclavine | Ergoline alkaloid | 1.8863 | up | down | 1.4831 | up | down | no |
| 38 | metab_24264 | Vanilloyl glucose | Phenolic glycoside | 1.865 | up | down | 1.6692 | down | up | no |
| 39 | metab_2079 | Bis-(acetyl)diaminopentane | Acetylated polyamine | 1.8627 | up | down | 2.1575 | up | down | yes |
| 40 | metab_9796 | 1-Nonadecanoyl-glycero-3-phosphoserine | Phospholipid | 1.8429 | up | down | 2.378 | up | down | no |
| 41 | metab_17180 | Dihydrocaffeic acid 3-O-glucuronide | Hydroxycinnamic acid glucuronide | 1.8381 | up | down | 1.9081 | down | up | yes |
| 42 | metab_18891 | 17beta-Estradiol 3-sulfate | Estrogen conjugate | 1.8311 | up | down | 2.4262 | up | down | no |
| 43 | metab_4695 | 11beta, 13-Dihydrolactucin | Sesquiterpene lactone | 1.8177 | up | down | 1.8588 | up | down | no |
| 44 | metab_2433 | Leu-Leu | Dipeptide | 1.8107 | up | down | 2.3354 | up | down | yes |
| 45 | metab_19795 | St(24:1_o3_g) | Sulfolipid | 1.7662 | up | down | 1.6239 | up | down | no |
| 46 | metab_4570 | (3b, 6b, 8a, 12a)-8, 12-Epoxy-7(11)-eremophilene-6, 8, 12-trimethoxy-3-ol | Epoxy eremophilene derivative | 1.7582 | up | down | 1.2417 | down | up | no |
| 47 | metab_23638 | P-cresol sulfate | Sulfated phenol derivative | 1.7346 | up | down | 1.0687 | up | down | yes |
| 48 | metab_12820 | 2-Octenedioic acid | Unsaturated dicarboxylic acid | 1.7106 | up | down | 1.437 | down | up | no |
| 49 | metab_17229 | 3-(3,5-dihydroxyphenyl)-1-propanoic acid sulfate | Sulfated hydroxy phenylpropanoic acid | 1.6758 | up | down | 1.6405 | down | up | no |
| 50 | metab_13779 | Pyrogallol | Trihydroxybenzene derivative | 1.6692 | up | down | 1.6378 | up | down | no |
| 51 | metab_1677 | Tyr-Gly | Dipeptide | 1.6687 | up | down | 1.4569 | down | up | yes |
| 52 | metab_23992 | Aspirin | Salicylate derivative | 1.6589 | up | down | 1.1278 | down | up | no |
| 53 | metab_19093 | Helenalin | Sesquiterpene lactone | 1.6405 | up | down | 1.3438 | up | down | no |
| 54 | metab_1263 | 3'-C-Ethynylcytidine | Nucleoside analog | 1.6319 | down | up | 1.633 | down | up | no |
| 55 | metab_2069 | Brivaracetam | Pyrrolidone derivative | 1.6297 | up | down | 2.1092 | up | down | no |
| 56 | metab_13702 | L-Leucine | Essential amino acid | 1.6223 | up | down | 1.9478 | up | down | no |
| 57 | metab_3845 | Prednisolone acetate | Corticosteroid ester | 1.6022 | up | down | 2.0312 | up | down | no |
| 58 | metab_19529 | Glycochenodeoxycholic acid | Bile acid conjugate | 1.5952 | up | down | 1.5957 | up | down | yes |
| 59 | metab_2066 | N-(3-acetamidopropyl)pyrrolidin-2-one | Pyrrolidinone derivative | 1.5574 | up | down | 2.0868 | up | down | no |
| 60 | metab_24403 | 2-(2-Hydroxyethyl)-4-oxohexanedioic acid | Keto dicarboxylic acid | 1.5424 | up | down | 2.1342 | down | up | no |
| 61 | metab_13485 | 4-amino-2-pyrazin-2-ylpyrimidine-5-carbonitrile | Aminopyrazinylpyrimidine derivative | 1.5368 | up | down | 2.2326 | up | down | no |
| 62 | metab_12959 | Actrarit | Amino benzoic acid derivative | 1.5363 | up | down | 1.9066 | down | up | no |
| 63 | metab_22295 | N-Choloylglycine | Bile acid conjugate | 1.5287 | up | down | 1.3656 | up | down | yes |
| 64 | metab_16477 | Isocitric acid | Tricarboxylic acid cycle intermediate | 1.5286 | up | down | 1.8425 | down | up | yes |
| 65 | metab_12699 | 5-(2-nitroprop-1-enyl)-1,3-benzodioxole | Nitrobenzodioxole derivative | 1.5253 | up | down | 1.1629 | down | up | no |
| 66 | metab_18791 | 1-(1-Hydroxybutyl)-1, 3, 4, 5, 6,7-hexahydro-2-benzofuran-4, 5, 6,7-tetrol | Hydroxybutyl benzofuran tetrol | 1.5243 | up | down | 1.1404 | down | up | no |
| 67 | metab_18472 | 3-Isochromanone | Isochromanone derivative | 1.5238 | up | down | 1.8236 | down | up | no |
| 68 | metab_2674 | 4,6-dihydroxy-3-methyl-2,3-dihydroisoindol-1-one | Dihydroxy methyl isoindolone | 1.5226 | up | down | 1.133 | down | up | no |
| 69 | metab_23606 | Formononetin-B-D-glucuronide sodium salt | Isoflavone glucuronide conjugate | 1.4966 | up | down | 1.663 | down | up | yes |
| 70 | metab_16073 | 3-(4-Ethyl-6-Sulfo-1, 2, 3,4-Tetrahydronaphthalen-1-yl)propanoic Acid | Sulfated tetrahydronaphthyl propanoic acid | 1.4681 | up | down | 1.1251 | down | up | no |
| 71 | metab_1476 | 5-Pyridoxolactone | Pyridoxine derivative | 1.4675 | up | down | 1.2481 | up | down | yes |
| 72 | metab_1794 | 6-Acetamido-6-deoxy-castanospermine | Acetylated castanospermine derivative | 1.4654 | up | down | 1.1003 | up | down | no |
| 73 | metab_12220 | Glucitol-lysine | Sugar amino acid conjugate | 1.464 | up | down | 2.1228 | down | up | no |
| 74 | metab_13021 | 2,4-quinolinediol | Quinoline derivative | 1.4443 | up | down | 1.7445 | up | down | no |
| 75 | metab_12380 | . beta. -(2,5-Dimethylbenzoyl)propionic acid | Benzoyl propionic acid derivative | 1.4378 | up | down | 1.5559 | down | up | no |
| 76 | metab_18591 | [[amino(phenyl)methylidene]amino] 5-tert-butyl-2-methylpyrazole-3-carboxylate | Pyrazole carboxylate derivative | 1.4311 | up | down | 1.5421 | up | down | no |
| 77 | metab_25341 | Citric acid | Tricarboxylic acid cycle intermediate | 1.4157 | up | down | 1.6634 | down | up | yes |
| 78 | metab_2170 | (2S)-2,5-Diamino-4-fluoro-5-oxopentanoic acid | Fluorinated amino acid derivative | 1.3763 | up | down | 1.6263 | down | up | no |
| 79 | metab_23532 | 6-Methoxymellein | Mellein derivative | 1.3316 | up | down | 1.3741 | down | up | no |
| 80 | metab_21600 | 5-(1,1-dimethylheptyl)-2-[5-hydroxy-2-(3-hydroxypropyl)cyclohexyl]phenol | Alkyl cyclohexyl phenol derivative | 1.3302 | down | up | 1.8773 | up | down | no |
| 81 | metab_12720 | 2-piperidinobenzoic acid | Piperidinyl benzoic acid derivative | 1.3088 | up | down | 1.584 | up | down | no |
| 82 | metab_17545 | Hydroxymedioresinol | Lignan | 1.3072 | up | down | 2.4776 | up | down | no |
| 83 | metab_25199 | 1, 2,3-cyclopropanetricarboxylic acid | Cyclopropane carboxylic acid derivative | 1.2916 | up | down | 1.9027 | down | up | no |
| 84 | metab_13222 | Ala-Gly-Ile | Tripeptide | 1.2794 | up | down | 1.3029 | down | up | yes |
| 85 | metab_24976 | Ile-Asp | Dipeptide | 1.259 | up | down | 1.7741 | down | up | yes |
| 86 | metab_680 | Homoarginine | Amino acid derivative | 1.2235 | up | down | 1.2773 | down | up | no |
| 87 | metab_18600 | Diethylpropion | Ketone derivative | 1.2085 | up | down | 1.6386 | up | down | no |
| 88 | metab_21693 | 8,9-ee-14(z)-e | Unsaturated fatty acid derivative | 1.2074 | down | up | 1.9047 | down | up | no |
| 89 | metab_24342 | Laccaic acid D | Anthraquinone derivative | 1.2064 | down | up | 2.0259 | up | down | no |
| 90 | metab_23016 | Abscisic acid | Plant hormone | 1.2026 | up | down | 1.1955 | up | down | no |
| 91 | metab_1062 | Pseudouridine | Modified nucleoside | 1.1897 | up | down | 1.3764 | down | up | no |
| 92 | metab_14335 | Histidinal | Histidine derivative | 1.1849 | up | down | 1.3131 | down | up | no |
| 93 | metab_22415 | Aleuretic acid | Ergot alkaloid derivative | 1.1821 | up | down | 1.1823 | down | up | no |
| 94 | metab_18296 | Dolichodial | Sesquiterpene dialdehyde | 1.1641 | up | down | 1.2993 | down | up | no |
| 95 | metab_12109 | 5-(2,5-Dihydroxyhexyl)oxolan-2-one | Hydroxyhexyl oxolanone | 1.1621 | up | down | 1.8312 | down | up | no |
| 96 | metab_16356 | Trans-aconitic acid | Tricarboxylic acid | 1.16 | up | down | 2.5354 | down | up | yes |
| 97 | metab_12110 | 4-tert-butylcatechol | Alkyl catechol derivative | 1.1511 | up | down | 1.2675 | down | up | no |
| 98 | metab_22553 | Riesling acetal | Acetal derivative | 1.124 | down | up | 1.8004 | down | up | no |
| 99 | metab_25616 | N-acetyl-d-galactosamine 4-sulfate | Sulfated amino sugar | 1.1139 | up | down | 2.7757 | down | up | no |
| 100 | metab_25623 | Allantoin | Purine metabolite | 1.1114 | up | down | 1.97 | up | down | yes |
| 101 | metab_23053 | Altamisic acid | Diterpenoid carboxylic acid | 1.1044 | up | down | 1.3104 | up | down | no |
| 102 | metab_26248 | Zeranol | Nonsteroidal estrogen | 1.0894 | up | down | 1.1465 | down | up | no |
| 103 | metab_22562 | 4-hydroxycoumarin | Coumarin derivative | 1.0765 | up | down | 1.9626 | up | down | no |
| 104 | metab_24081 | N-(2-Furoyl)glycine | Furoyl amino acid conjugate | 1.0759 | up | down | 1.8472 | down | up | yes |
| 105 | metab_11541 | 5-(4-hydroxypentyl)benzene-1,3-diol | Hydroxypentyl benzene diol | 1.0674 | up | down | 1.3132 | down | up | no |
| 106 | metab_24319 | Daphnoretin | Coumarin derivative | 1.0592 | down | up | 1.6206 | up | down | no |
| 107 | metab_25501 | D-(-)-Ribose | Pentose monosaccharide | 1.0438 | up | down | 1.3156 | down | up | no |
| 108 | metab_16049 | 5-Keto-D-Gluconate | Keto gluconate derivative | 1.042 | up | down | 1.6187 | down | up | yes |
| 109 | metab_3066 | (2E, 4E)-2,7-dimethylocta-2,4-dienedioic acid | Unsaturated dicarboxylic acid | 1.0372 | up | down | 1.1818 | up | down | no |
| 110 | metab_19684 | 13, 14-dihydro-15-keto-tetranor Prostaglandin D2 | Prostanoid metabolite | 1.0367 | up | down | 1.2151 | down | up | no |
| 111 | metab_1048 | FAPy-adenine | Modified adenine derivative | 1.0321 | up | down | 1.4474 | up | down | no |
| 112 | metab_4812 | Traumatic acid | Dicarboxylic acid | 1.0159 | up | down | 1.0925 | down | up | no |
| 113 | metab_19582 | 2-octylpent-2-enedioic acid | Alkyl unsaturated dicarboxylic acid | 1.0097 | up | down | 1.0017 | down | up | no |
